# Host cell death during infection with *Chlamydia*: a double-edged sword

**DOI:** 10.1093/femsre/fuaa043

**Published:** 2020-09-08

**Authors:** Barbara S Sixt

**Affiliations:** The Laboratory for Molecular Infection Medicine Sweden (MIMS), Umeå Centre for Microbial Research (UCMR), Department of Molecular Biology, Umeå University, SE-901 87 Umeå, Sweden

**Keywords:** intracellular bacteria, virulence strategies, regulated cell death, bacterial exit, bacterial toxicity, cell-autonomous immunity

## Abstract

The phylum *Chlamydiae* constitutes a group of obligate intracellular bacteria that infect a remarkably diverse range of host species. Some representatives are significant pathogens of clinical or veterinary importance. For instance, *Chlamydia trachomatis* is the leading infectious cause of blindness and the most common bacterial agent of sexually transmitted diseases. *Chlamydiae* are exceptionally dependent on their eukaryotic host cells as a consequence of their developmental biology. At the same time, host cell death is an integral part of the chlamydial infection cycle. It is therefore not surprising that the bacteria have evolved exquisite and versatile strategies to modulate host cell survival and death programs to their advantage. The recent introduction of tools for genetic modification of *Chlamydia* spp., in combination with our increasing awareness of the complexity of regulated cell death in eukaryotic cells, and in particular of its connections to cell-intrinsic immunity, has revived the interest in this virulence trait. However, recent advances also challenged long-standing assumptions and highlighted major knowledge gaps. This review summarizes current knowledge in the field and discusses possible directions for future research, which could lead us to a deeper understanding of *Chlamydia*’s virulence strategies and may even inspire novel therapeutic approaches.

## Teaser

A comprehensive review that highlights recent advances and major knowledge gaps in our understanding of the diverse mechanisms and roles of pathogen-mediated host cell death modulation during infection with the obligate intracellular pathogen *Chlamydia trachomatis* and its relatives.

## INTRODUCTION

The phylum *Chlamydiae* is a group of obligate intracellular bacteria that infect a wide range of host species, including members of all major groups of vertebrates, as well as invertebrates, and even unicellular eukaryotes (Horn 2008). From a medical perspective, the most important representatives are species in the genus *Chlamydia*. For instance, *Chlamydia trachomatis* (serovars A–C) is the causative agent of trachoma, an ocular disease that is the leading infectious cause of blindness (Taylor *et al*. 2014). *Chlamydia trachomatis* (serovars D–K) is also the most frequent bacterial agent of sexually transmitted diseases and as such a significant cause of infertility and adverse pregnancy outcomes (Newman *et al*. 2015). Moreover, certain sexually transmitted strains of *C. trachomatis* (serovars L1–L3) can cause lymphogranuloma venereum (LGV) (Ceovic and Gulin 2015). *Chlamydia pneumoniae*, the second major human pathogenic *Chlamydia* species, primarily causes respiratory tract infections, yet was also proposed to be associated with atherosclerosis and neurological disorders (Burillo and Bouza 2010). Other *Chlamydia* species, such as *Chlamydia psittaci*, infect primarily animals, but can occasionally cause severe zoonotic infections (Longbottom and Coulter 2003). Moreover, some animal pathogenic species, such as the mouse pathogen *Chlamydia muridarum* and the guinea pig pathogen *Chlamydia caviae*, are frequently used as models in research, due to the availability of convenient *in vivo* infection models (Rank 2012). Chlamydial species within other genera and families in the phylum *Chlamydiae* are often collectively referred to as environmental chlamydiae (Horn 2008). While recent studies uncovered an astonishing diversity in this group, their biology and impact are less well explored (Collingro, Köstlbacher and Horn 2020). It is clear that a better understanding of these microbes could provide valuable insights into the evolution of virulence strategies in *Chlamydiae* and their role in host adaptation (Horn 2008). Although this review will primarily focus on species in the genus *Chlamydia*, it will therefore also summarize available data on cultured environmental species.

**Figure 1.**
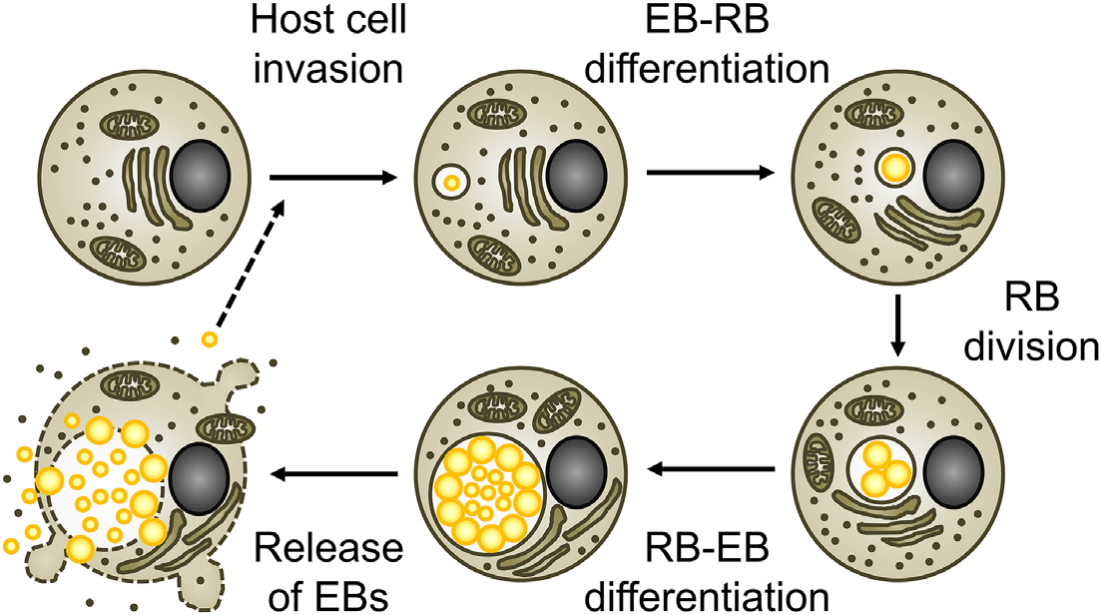
The chlamydial developmental cycle. Schematic representation of the different stages of the *Chlamydia* developmental cycle. Bacteria are indicated as yellow spheres. Small and large spheres represent EBs and RBs, respectively.

All cultured *Chlamydiae* share a unique lifestyle that is characterized by a strict dependence on a eukaryotic host cell and a biphasic developmental cycle (Ward 1988) (Fig. 1). The infective developmental form, called the elementary body (EB), invades the host cell in a process that leads to the generation of a membrane-bound pathogen-containing vacuole, named the inclusion. Within the inclusion, the EB differentiates into the replicative form, the reticulate body (RB). Due to their adaptation to the intracellular growth niche, *Chlamydiae* have highly reduced metabolic capacities (Stephens *et al*. 1998; Collingro *et al*. 2011; Omsland *et al*. 2014). The bacteria evolved to redirect nutrients and lipids from host cell compartments to the inclusion (Saka and Valdivia 2010). RBs are also energy parasites that can directly feed on the host cell's pool of adenosine triphosphate (ATP) (Hatch, Al-Hossainy and Silverman 1982), although the bacteria also have the capacity to generate ATP on their own (Omsland *et al*. 2012). *Chlamydiae* actively modulate host cellular functions, in part by exploiting their type III secretion system to deliver effector proteins into the host cell cytosol (Peters *et al*. 2007). A special class of these effectors, the inclusion membrane proteins (Incs), is inserted into the membrane of the inclusion (Rockey and Alzhanov 2006). After several rounds of division, RBs asynchronously re-differentiate into EBs, which are eventually released from the host cell to infect neighboring cells (Hybiske and Stephens 2007). The length of this developmental cycle can be variable, but typically ranges from 36 to 96 h for *Chlamydia* spp. in cultured cells (Miyairi *et al*. 2006).

As a consequence of this unique lifestyle, *Chlamydiae* are absolutely dependent on the integrity of their host cell until they have completed their developmental cycle, i.e. until a significant number of bacteria have re-differentiated into mature EBs. During the past decades, the interaction of pathogenic *Chlamydia* spp. with their host cells’ death and survival pathways has been the subject of extensive investigations. While these studies revealed a complex picture in which *Chlamydia* spp. both induced and blocked host cell death, they particularly highlighted the pronounced anti-apoptotic trait of the pathogen (Ying *et al*. 2007; Sharma and Rudel 2009). However, recent findings challenged the effectiveness of *Chlamydia*’s anti-apoptotic activities as means to preserve host cell viability (Sixt *et al*. 2018). At the same time, advances in our ability to genetically modify *Chlamydia* spp. (Sixt and Valdivia 2016) revealed cell death as a host cellular defense response that could be effective, but is actively suppressed by the pathogen (Sixt *et al*. 2017; Weber *et al*. 2017; Giebel *et al*. 2019). A profound understanding of this complex interplay between host and pathogen is critical for our understanding of *Chlamydia* diseases and anti-chlamydial host defenses. In the future, it may even enable us to exploit *Chlamydia*’s interference with cell death as target for novel anti-chlamydial treatment strategies.

This review aims to provide a comprehensive and structured overview of current knowledge, with focus on recent advances, to establish links between distinct facets of *Chlamydia*-mediated cell death modulation, and to highlight knowledge gaps. Its objective is to stimulate cross-disciplinary discussions and to inspire future research.

## MODES OF CELL DEATH

### Accidental and regulated forms of cell death

Extreme physical, mechanical or chemical insults on eukaryotic cells, for example high temperatures, shear stress or extreme pH variations, can cause the instantaneous death of the cell. Such accidental cell death (ACD) does not depend on the activity of cellular regulators (Galluzzi *et al*. 2018). As counter pole to ACD, the term programmed cell death (PCD) was first introduced in 1964 to describe the observation that in multicellular organisms certain cells seemed to be programmed to die at specific stages during development (Lockshin 1964). However, genetically encoded cell-intrinsic death programs, which govern PCD, act in not only such developmentally programmed events but also cell death that is induced in response to severe non-compensable perturbations of the cellular homeostasis. Therefore, the more general term regulated cell death (RCD) was introduced more recently to encompass all forms of cell death that result from the activation of molecular death machineries (Galluzzi *et al*. 2018).

### Morphology of cell death

**Figure 2.**
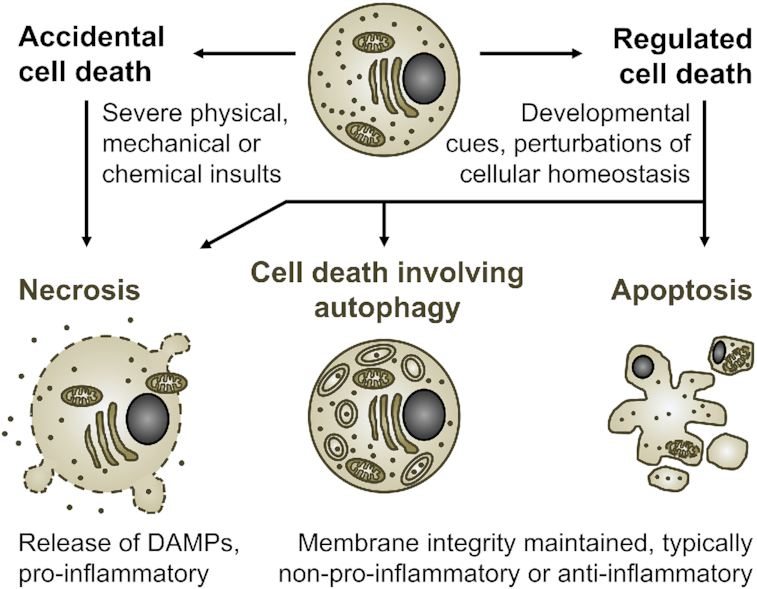
Inducers, cell morphologic presentation, and inflammatory potential of accidental and regulated cell death. Note that immunogenic forms of apoptosis, which are accompanied by release of DAMPs, have also been described. Moreover, under conditions in which phagocytic removal of dying cells is insufficient, even cells that initiate non-necrotic forms of cell death will eventually become secondary necrotic.

Morphologically, ACD is a form of necrosis, characterized by cell swelling and a sudden rupture of the plasma membrane (Proskuryakov, Konoplyannikov and Gabai 2003). Based on the characteristics of cells dying from PCD, two major forms of cell death distinct from necrosis were described: cell death involving autophagy, characterized by extensive cytoplasmic vacuolization, and apoptosis, involving cell shrinkage, membrane blebbing, nuclear condensation and cell fragmentation (Kerr, Wyllie and Currie 1972; Schweichel and Merker 1973). However, the increasing focus on molecular determinants for the classification of cell death, as opposed to morphological traits, and the developing awareness that RCD serves functions beyond development have led to the recognition of several modes of regulated necrosis, such as necroptosis (Degterev *et al*. 2005) and pyroptosis (Boise and Collins 2001; Cookson and Brennan 2001), that are executed by a molecular suicide machinery, but share morphological characteristics with ACD (Fig. 2).

### Inflammatory potential and immunogenicity of cell death

The way in which a cell dies has a strong impact on how it is perceived by surrounding cells and by the immune system (Galluzzi *et al*. 2017). Necrotic forms of cell death lead to the release of cellular content into the surroundings of the dying cell. Among the molecules released are those that act as danger-associated molecular patterns (DAMPs), for instance ATP and high mobility group protein B1 (HMGB1) (Kono and Rock 2008). The pro-inflammatory environment that is thereby generated supports the development of immune responses against antigens present in the dying cells (Galluzzi *et al*. 2017). In contrast, under physiological conditions, the plasma membrane integrity of cells dying by apoptosis or cell death involving autophagy is maintained until the cells are phagocytosed and degraded (Kerr, Wyllie and Currie 1972; Schweichel and Merker 1973). This enables a safe and usually immunologically silent removal. However, immunogenic forms of apoptosis, which are accompanied by release of DAMPs, have been described (Galluzzi *et al*. 2017). Moreover, under conditions in which phagocytic removal of dying cells is insufficient, apoptotic cells will eventually become secondary necrotic (Silva, do Vale and dos Santos 2008).

### Molecular basis of selected death programs

#### Apoptosis

**Figure 3.**
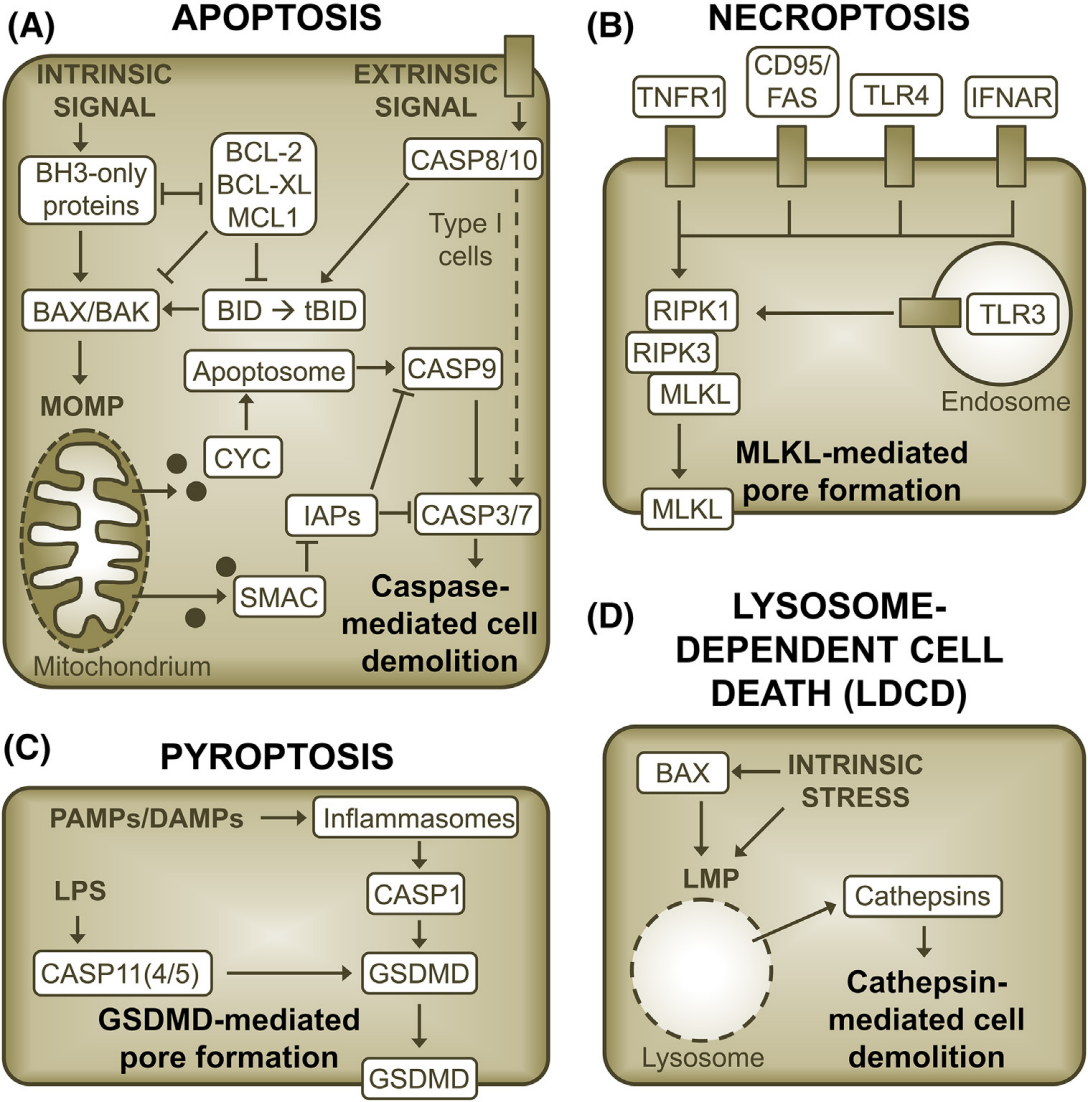
The molecular basis of selected cell death programs. Illustration of key events in apoptosis **(A)**, necroptosis **(B)**, pyroptosis **(C)** and lysosome-dependent cell death **(D)**. The representations are simplified. Cross-talks between pathways and links to inflammatory signaling are not depicted. Moreover, in (B), the signaling events between the receptors and the effectors of necroptosis are not shown. RIPK1 is dispensable for some modes of necroptosis.

Two major pathways of apoptotic signaling can be distinguished: intrinsic and extrinsic apoptosis (Elmore 2007) (Fig. 3A). Intrinsic apoptosis can result from a variety of microenvironmental perturbations, such as growth factor withdrawal, DNA damage or mitotic defects (Galluzzi *et al*. 2018). A central step in the intrinsic pathway is the mitochondrial outer membrane permeabilization (MOMP) (Galluzzi, Kepp and Kroemer 2016). MOMP causes the release of mitochondrial cytochrome c (CYC), followed by the formation of a cytosolic signaling complex that leads to the activation of the initiator caspase caspase-9 (CASP9) (Bao and Shi 2007). CASP9 in turn mediates the activation of effector caspases [caspase-3 (CASP3) and caspase-7 (CASP7)], whose proteolytic action initiates cell demolition (Kumar 1999). MOMP is mediated by the activity of the pro-apoptotic B-cell lymphoma 2 (BCL-2) family proteins BCL-2-associated X protein (BAX) and BCL-2-antagonist killer (BAK), which is tightly regulated by pro-apoptotic BH3-only proteins and anti-apoptotic BCL-2 family proteins (Czabotar *et al*. 2014). In the extrinsic pathway, engagement of death receptors, such as Fas receptor (CD95/FAS) or tumor necrosis factor receptor 1 (TNFR1), can lead to the formation of a signaling complex that activates the initiator caspase caspase-8 (CASP8) (Guicciardi and Gores 2009). Depending on the cell type, the proteolytic activity of CASP8 may be sufficient to directly activate effector caspases resulting in cell death (type I cells) or induction of cell death may require an amplification of pro-death signaling via CASP8-mediated cleavage of BH3-interacting domain death agonist (BID) and subsequent induction of MOMP (type II cells) (Kantari and Walczak 2011).

#### Necroptosis

Necroptosis is a necrotic mode of RCD that can be activated downstream of certain death receptors, such as CD95/FAS and TNFR1, and pathogen recognition receptors, such as Toll-like receptor 3 (TLR3) and Toll-like receptor 4 (TLR4) (Degterev *et al*. 2005; Pasparakis and Vandenabeele 2015) (Fig. 3B). In the context of TNFR1 signaling, necroptosis is considered a back-up for apoptosis, because it is only activated under circumstances in which CASP8 is absent or its activity blocked (Degterev *et al*. 2005; Tummers and Green 2017). Mechanistically, necroptosis induction depends on the activation of receptor-interacting serine/threonine-protein kinase 3 (RIPK3), which phosphorylates mixed lineage kinase domain-like protein (MLKL), resulting in the formation of MLKL oligomers that induce plasma membrane permeabilization (Sun *et al*. 2012b; Cai *et al*. 2014). During tumor necrosis factor alpha (TNFα)-induced necroptosis, RIPK3 activation depends on receptor-interacting serine/threonine-protein kinase 1 (RIPK1) and can hence be blocked by inhibitors of RIPK1 (Degterev *et al*. 2008). However, RIPK1 is not involved in all types of necroptotic signaling (Pasparakis and Vandenabeele 2015).

#### Pyroptosis

Pyroptosis is a form of regulated necrosis that depends on the activation of one or more inflammatory caspases (Cookson and Brennan 2001; Jimenez Fernandez and Lamkanfi 2015) (Fig. 3C). These enzymes are activated in response to pathogen-associated molecular patterns (PAMPs) or DAMPs (Jimenez Fernandez and Lamkanfi 2015). While murine caspase-11 (CASP11) [and human caspase-4 (CASP4) and caspase-5 (CASP5)] can directly respond to bacterial lipopolysaccharide (LPS) (Shi *et al*. 2014), caspase-1 (CASP1) is activated indirectly via inflammasomes (Martinon, Burns and Tschopp 2002). Once activated beyond a critical threshold, inflammatory caspases catalyze the proteolytic cleavage of gasdermin D (GSDMD) (Kovacs and Miao 2017). The N-terminal fragment of GSDMD can then oligomerize at the plasma membrane, resulting in pore formation and cell death (Kovacs and Miao 2017). Because of the involvement of inflammatory caspases, pyroptosis is usually accompanied by secretion of interleukin 1 beta (IL-1β) and interleukin 18 (IL-18) and hence mediates robust pro-inflammatory effects (Jimenez Fernandez and Lamkanfi 2015).

#### Lysosome-dependent cell death

Soon after their discovery in the 1950s, lysosomes were dubbed cellular 'suicide bags', because it was suggested that lysosomal rupture could be a major mechanism of RCD (De Duve 1959). While the role of lysosome-dependent cell death (LDCD) may not be as fundamental as initially suggested, examples exist in which intracellular perturbations, such as oxidative stress or disruption of the cytoskeleton, can cause lysosomal membrane permeabilization (LMP) (Aits *et al*. 2015; Wang, Gomez-Sintes and Boya 2018) (Fig. 3D). LMP can also be mediated by BAX, which in some instances may precede BAX-mediated MOMP (Kagedal *et al*. 2005; Bove *et al*. 2014; Guan *et al*. 2015). The proteolytic enzymes of the cathepsin family, which are released from lysosomes during LMP, are considered main executors of LDCD, because inhibition of cathepsin activity can in many instances ameliorate LDCD (Wang, Gomez-Sintes and Boya 2018). However, the mode of cell death induced by LMP also depends on its extent. While massive LMP appears to induce rapid necrotic death, partial LMP may for instance induce MOMP and apoptosis or inflammasome activation and pyroptosis (Wang, Gomez-Sintes and Boya 2018). Moreover, in some instances, LMP does not initiate cell death by itself, but accompanies and amplifies other death signals, such as during certain instances of apoptosis (Galluzzi *et al*. 2018).

### Roles of cell death during infection

**Figure 4.**
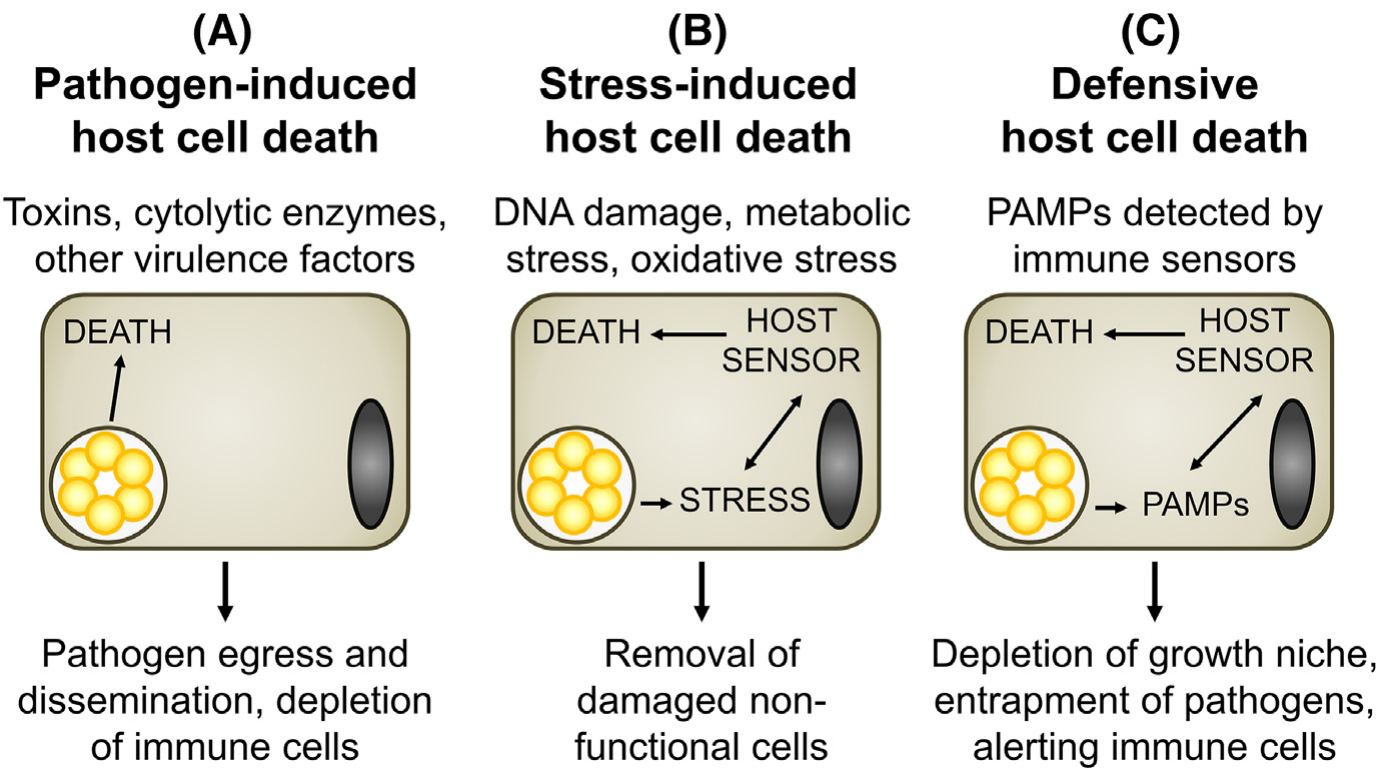
The various roles of infection-associated cell death. **(A)** Host cell death can be triggered by the pathogen, for instance to mediate pathogen release, spread to deeper tissue layers, or immune cell depletion. **(B)** Host cell death may be triggered by the host cell itself as response to non-compensable infection-induced stress, such as DNA damage, oxidative stress or metabolic stress. **(C)** Upon detection of an invading pathogen by immune sensors, host cells may trigger cell death as defense response. Note that immune mediators, such as cytotoxic cytokines and immune cell-mediated killing mechanisms, can promote host cell death either by inducing cellular stress (B) or by supporting death-inducing host cell-intrinsic defense responses (C).

It is well known that the encounter of eukaryotic cells with extracellular pathogens or the invasion by intracellular pathogens can eventually result in the death of the cell. Different scenarios of infection-associated cell death can be distinguished (Fig. 4). First, cell death may be mediated by a pathogen-driven process, such as by the action of toxins or cytolytic enzymes, for instance to enable release of intracellular pathogens, to facilitate pathogen dissemination or to mediate depletion of immune cells (Fig. 4A). Pathogen-driven host cell death depends on virulence factors and may or may not rely on the (partial) induction of host RCD programs. Second, cell death may be induced by host cell-intrinsic mechanisms in response to microenvironmental perturbations caused by the presence of the pathogen, such as nutrient deprivation, DNA damage or oxidative stress (Fig. 4B). Third, cell death may be triggered by the host cell as a defense response, for example to restrict intracellular pathogen replication by removing the replicative niche, to entrap the pathogen to limit its dissemination, to alert neighboring cells and immune cells, and/or to shape the subsequent immune response against the pathogen (Fig. 4C). Both forms of host cell-driven cell death, stress-induced and defensive, may be actively counteracted by the pathogen. Immune mediators, such as cytotoxic cytokines and cell-mediated killing mechanisms, can promote host cell death either by inducing cellular stress or by supporting death-inducing cell-intrinsic defense responses. Given these complex implications of host cell death, it is not surprising that a pathogen that is as dependent on its host cell as *Chlamydia* has evolved both pro- and anti-death activities, which can be variably active under distinct circumstances.

## PRO-DEATH ACTIVITIES OF CHLAMYDIAE

### Host cell death as exit strategy

#### Cell death is an integral part of the Chlamydia infection cycle

Host cell death has long been recognized as the final stage of the *Chlamydia* infection cycle, enabling the release of EBs and spread of infection. The *Chlamydia* developmental cycle was first described for *C. psittaci* in 1932 based on light microscopic observations (Bedson and Bland 1932). Subsequently, electron microscopic analyses gave deeper insights into the morphological differences between EBs and RBs and their relation to each other (Swain 1955; Tajima, Nomura and Kubota 1957). In the 1960s, studies in cell culture revealed that infectivity was lost after the invading EBs had differentiated into RBs and reappeared once RBs had re-differentiated into EBs (Bernkopf, Mashiah and Becker 1962; Higashi, Tamura and Iwanaga 1962). Moreover, host cell death was observed at late stages and was accompanied by an increase of infectivity in culture supernatants (Higashi, Tamura and Iwanaga 1962; Friis 1972; Todd and Storz 1975). It can be expected that the mode by which host cells release EBs greatly impacts the magnitude of tissue damage and inflammation at the infection site. It will also shape the nature of the subsequent immune response and will affect the viability and spreading potential of the released bacteria. Astonishingly, our knowledge of the molecular events that govern *Chlamydia* egress is still scarce.

#### The role of lysosomes in Chlamydia exit

Several studies conducted in the 1970s suggested that *Chlamydia* exit could be preceded by LMP (Kordova, Wilt and Sadiq 1971; Kordova and Wilt 1972; Kordova, Hoogstraten and Wilt 1973; Todd and Storz 1975). Cell fractionation experiments and ultrastructural protein localization studies demonstrated that in bovine cells infected with *Chlamydia pecorum*, enzymes were released from lysosomes prior to host cell death (Todd and Storz 1975). LMP also appeared to precede cell death in murine fibroblasts and macrophages infected with *C. psittaci* (Kordova, Wilt and Sadiq 1971; Kordova and Wilt 1972; Kordova, Hoogstraten and Wilt 1973). Interestingly, these observations were not followed up upon in more recent studies. A critical reevaluation of a potential role of LMP in *Chlamydia* exit using more advanced tools would be timely.

#### The role of the apoptotic program in Chlamydia exit

In 1998, Ojcius and colleagues reported that cell death induced by *C. caviae* in human epithelial (HeLa) cells and murine macrophages was accompanied by features that were characteristic for apoptosis (Ojcius *et al*. 1998). One of these features was DNA fragmentation, which was detected by various techniques, such as the terminal deoxynucleotidyl transferase dUTP nick end labeling (TUNEL) assay, which enables microscopic detection of DNA strand breaks, the DNA ladder assay, which enables the display of nucleosomal DNA fragmentation on agarose gels, and flow cytometry of propidium iodide-stained permeabilized cells, which enables the detection of cells with sub-diploid DNA content (Ojcius *et al*. 1998). Furthermore, ultrastructural changes indicative for apoptosis, such as cell shrinkage and chromatin condensation, were observed (Ojcius *et al*. 1998). DNA fragmentation and nuclear condensation were also detected in cells infected with *C. psittaci*, *C. muridarum* or *C. trachomatis* L2 (Gibellini, Panaya and Rumpianesi 1998; Perfettini *et al*. 2000; Perfettini *et al*. 2002b; Ying *et al*. 2006). Moreover, TUNEL-positive cells were detected in the genital tract tissue of mice infected with *C. muridarum* (Perfettini *et al*. 2000). Finally, the surface of mouse embryonic fibroblasts (MEFs) that died after infection with *C. muridarum* or *C. trachomatis* L2 could be stained with fluorescent Annexin V (ANX5) (Perfettini *et al*. 2003a; Jungas *et al*. 2004). This is a marker for apoptosis, because ANX5 binds phosphatidylserine, a lipid that typically resides in the inner leaflet of the plasma membrane, but in apoptotic cells is translocated to the outer leaflet to trigger phagocytic removal of the dying cell (Koopman *et al*. 1994). In some studies, the use of microscopic assays also confirmed that the above-mentioned apoptosis-like features occurred indeed in cells that contained *Chlamydia* inclusions (Gibellini, Panaya and Rumpianesi 1998; Ojcius *et al*. 1998; Perfettini *et al*. 2000; Ying *et al*. 2006).

Although the features described above are generally considered hallmarks of caspase-dependent apoptosis, these studies also reported that cell death induced by *C. caviae* or *C. trachomatis* L2 could not be blocked by a specific inhibitor of CASP3 nor by the pan caspase inhibitor Z-VAD-FMK, suggesting that it was a caspase-independent form of cell death (Ojcius *et al*. 1998; Perfettini *et al*. 2002b; Ying *et al*. 2006). Indeed, no activation of apoptotic effector caspases could be observed in late stage infected cells (Perfettini *et al*. 2002b; Ying *et al*. 2006). The reliability of ANX5 staining for detection of apoptosis in *Chlamydia-*infected cells is also questionable, because *Chlamydia* infection itself can induce phosphatidylserine externalization in the absence of an activation of the apoptotic program (Galle *et al*. 2019). Moreover, DNA ladders induced by *C. trachomatis* L2 were atypical and it was therefore proposed that the mechanism of DNA fragmentation in infected cells was different from that that operates during apoptosis (Ying *et al*. 2006). In disagreement with the above-mentioned findings, a recent study reported that cell death induced at late stage of infection with *C. trachomatis* (L2 or D) can be blocked or delayed by Z-VAD-FMK and is accompanied by effector caspase activity (Foschi *et al*. 2019). In this study, cell death was monitored at the cell population level and the occurrence of apoptotic features was not directly demonstrated in inclusion-containing cells.

Interestingly, BAX activation was detected in HeLa cells infected with *C. caviae* or *C. muridarum*, but not after infection with *C. trachomatis* L2, and in the former cells activated BAX appeared to co-localize with mitochondria (Perfettini *et al*. 2002b; Jungas *et al*. 2004). Furthermore, overexpression of the BAX inhibitor 1 (BI-1) or the anti-apoptotic protein BCL-2 significantly reduced the occurrence of apoptotic nuclear morphology in *C. caviae*-infected cells (Perfettini *et al*. 2002b). A reduction in 'apoptotic' cell death, inferred from reduced emergence of cells with sub-diploid DNA content or nuclear condensation, was also observed during *C. muridarum* or *C. trachomatis* L2 infection in BAX- or BAK-deficient MEFs when compared with wild-type cells (Perfettini *et al*. 2003a; Ying *et al*. 2006). However, no release of mitochondrial CYC was observed in infected wild-type MEFs (Ying *et al*. 2006), which together with the caspase-independent nature of *Chlamydia*-induced cell death indicated that the proposed pro-death role of these proteins during infection must differ from their usual role in apoptotic signaling. It is possible that in the context of infection, BAX and BAK may contribute to cell death by inducing LMP instead of MOMP. But further work is needed to test this hypothesis experimentally.

In cultures of BAX-deficient MEFs, spread of *C. muridarum* infection to formerly uninfected cells appeared to be impeded (Perfettini *et al*. 2003a). Moreover, in absence of BAX, infected MEFs appeared to die more frequently by necrosis (Perfettini *et al*. 2003a). Consistently, in BAX-deficient mice, infection was cleared faster, but signs of enhanced inflammation and tissue damage were observed (Perfettini *et al*. 2003a). Based on these findings, it was suggested that BAX-mediated host cell death, compared with host cell necrosis, provides a more efficient and silent mode of spread by promoting uptake of dying infected cells through phagocytosis and by avoiding release of DAMPs (Perfettini *et al*. 2003a).

It is important to mention that the above-described 'apoptotic' features were not observed in all instances of late stage *Chlamydia-*mediated cell death. Depending on the combination of host cell type and *Chlamydia* strain studied, the default mode could also be necrotic. For instance, Jungas et al reported that in contrast to their observations made in MEFs, in HeLa cells, *C. trachomatis* L2 and *C. muridarum* induced a necrotic mode of host cell death that resulted in the release of HMGB1 (Jungas *et al*. 2004). Native egress of *C. trachomatis* L2 from HeLa cells was also not affected by BAX/BAK-deficiency (Kerr *et al*. 2017).

The variable nature of the findings described above, suggests that the question if components of the apoptotic machinery take part in *Chlamydia* exit may not have a simple answer. Because some of the observations made may represent unnatural behavior of immortalized cell lines or may have been influenced by the use of cells from non-matched host species, future studies, including mechanistic studies such as those described below, should preferentially focus on more natural infection systems, for example by using primary cells or tissues derived from the natural host species and infection site. Moreover, the possibility that distinct *Chlamydia* species use different exit strategies should not be neglected.

#### Late stage host cell death is a pathogen-triggered process

In 2007, Hybiske and Stephens developed an elegant approach to monitor the fate of individual infected HeLa cells by live cell microcopy (Hybiske and Stephens 2007). More specifically, the use of a cell line that expressed cytosolic green fluorescent protein (GFP) enabled the authors to detect host cell lysis as efflux of GFP from the host cell into the medium. Moreover, inclusion rupture was detectable as influx of GFP into the inclusion lumen. Using this approach, the authors demonstrated that late stage host cell death was a sequential inside-out process that was initiated by the rupture of the inclusion membrane, followed by the rupture of other intracellular structures, such as the nuclear envelope, and completed with the rupture of the host plasma membrane (Hybiske and Stephens 2007). The entire process was completed within ∼20 min. Morphological features characteristic for apoptosis were not observed (Hybiske and Stephens 2007). Protease inhibitors, in particular the cysteine protease inhibitor E64, blocked inclusion rupture and significantly delayed host cell death. In contrast, host plasma membrane rupture could be blocked by inhibition of calcium signaling (Hybiske and Stephens 2007).

The observed order of membrane rupture events led Hybiske and Stephens suggest that at least the initial step, the inclusion rupture, was triggered by the bacteria (Hybiske and Stephens 2007). Further support for an active contribution of the bacteria came from a recent study that demonstrated that the lytic exit of *C. trachomatis* L2 from HeLa cells was blocked or delayed when chloramphenicol, an inhibitor of bacterial protein synthesis, was added at a late stage of infection (Yang *et al*. 2015). Moreover, experiments with *C. trachomatis* L2 mutants that were deficient for the protease *Chlamydia* proteasome-like activity factor (CPAF) or for plasmid-encoded PGP4, suggested that these virulence factors contribute to host cell lysis (Yang *et al*. 2015). The underlying mechanisms are unclear, but it was suggested that PGP4, likely non-directly, promotes inclusion rupture by destabilizing the actin cage that provides the inclusion with mechanical support (Yang *et al*. 2015). While CPAF is known to cleave the intermediate filament protein vimentin, which could further destabilize the cytoskeletal support of the inclusion (Kumar and Valdivia 2008), recent work demonstrated that CPAF-dependent vimentin cleavage in infected cells occurs only post-inclusion lysis and that inclusion lysis was observed also during infection with a CPAF-deficient mutant (Snavely *et al*. 2014). Moreover, CPAF cannot represent the protease that Hybiske and Stephens found to contribute to inclusion rupture (Hybiske and Stephens 2007), because CPAF is a serine protease that is insensitive to the E64 protease inhibitor (Paschen *et al*. 2008).

Another *Chlamydia* factor that was proposed to contribute to inclusion and/or host plasma membrane lysis is the *C. trachomatis* protein CT153, which contains a membrane attack complex/perforin (MACPF) domain and was proposed to have pore-forming activity (Taylor *et al*. 2010; Taylor and Nelson 2014). The absence of functional CT153 orthologs from genomes of multiple *Chlamydia* strains, including strains of *C. pneumoniae*, *C. caviae* and *C. abortus* (Taylor and Nelson 2014), precludes a universal role of the MACPF domain-containing protein in *Chlamydia* exit. However, the recent generation of MACPF domain-containing protein deficient transposon insertion mutants, both in *C. trachomatis* and *C. muridarum* (LaBrie *et al*. 2019; Wang *et al*. 2019), now provides the opportunity for experimental clarification of its role in these species.

#### Late stage host cell death partially represents a host cell-driven process

To study exit of *C. trachomatis* L2 from infected HeLa cells, Kerr and colleagues developed an approach that was similar to that described before by Hybiske and Stephens and led to comparable observations, although nuclear disruption was not observed (Kerr *et al*. 2017). The authors moreover used multiphoton ablation to selectively rupture chlamydial inclusions inside infected cells (Kerr *et al*. 2017). This procedure revealed that inclusion rupture at any studied time point during infection resulted in subsequent host plasma membrane rupture, which, like native egress (Hybiske and Stephens 2007), was dependent on intracellular calcium signaling (Kerr *et al*. 2017). Laser-assisted inclusion rupture at earlier time points resulted in a slower progression to plasma membrane rupture than late stage inclusion disruption, suggesting that higher bacterial load may accelerate the process. Because chloramphenicol treatment and inhibition of CPAF did not affect ablation-triggered cell death, the authors suggested that the second step of host cell lysis, the rupture of the plasma membrane, was a host cell-driven process (Kerr *et al*. 2017). Moreover, the authors suggested that host calpains might contribute to the first step of host cell lysis, inclusion rupture, because inclusion rupture was delayed in presence of calpain inhibitors (Kerr *et al*. 2017). A requirement for host factors in lytic exit was further supported by the observation that cycloheximide (CHX), an inhibitor of host protein synthesis, could block or delay the natural egress of *C. psittaci* and *C. trachomatis* L2 from epithelial cells (Gibellini, Panaya and Rumpianesi 1998; Yang *et al*. 2015). However, another study reported that CHX had no significant effect on late stage cell death induced by *C. caviae* (Ojcius *et al*. 1998).

Cell death induced by laser ablation of inclusions was not accompanied by the activation of apoptotic caspases and could not be blocked by BAX/BAK-deficiency or Z-VAD-FMK (Kerr *et al*. 2017). Cell death was also unaffected by the CASP1 inhibitor VX765, RIPK1-deficiency or the RIPK1 inhibitor necrostatin-1 (Kerr *et al*. 2017). However, because necroptosis can proceed in absence of RIPK1 (Pasparakis and Vandenabeele 2015) and pyroptosis can be induced by other inflammatory caspases not all of which are efficiently blocked by Z-VAD-FMK (Chauvier *et al*. 2007; Jimenez Fernandez and Lamkanfi 2015), further experimentation is needed to clearly exclude an involvement of the necroptotic and pyroptotic pathways. It also remains an intriguing idea that *Chlamydia* spp. may co-opt pre-existing host cellular defense programs to trigger their exit from their host cells, as discussed below.

#### Alternative host cell fates

**Figure 5.**
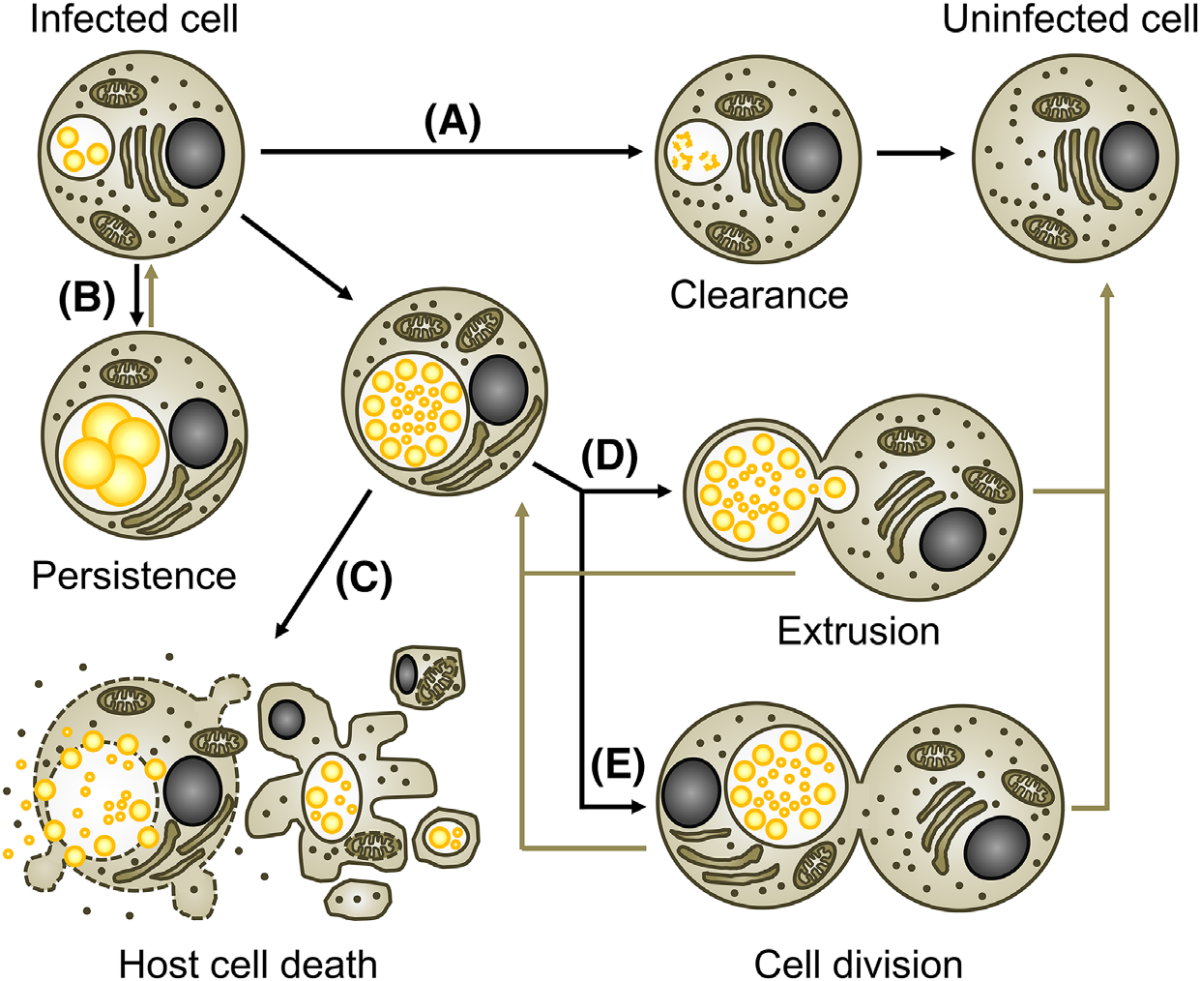
Alternative fates of *Chlamydia*-infected cells. The encounter with *Chlamydia* does not always lead to the death of the infected cell. **(A)** Under certain conditions, in particular after invasion of a professional phagocyte, *Chlamydia* infection can be cleared by the host cell, for instance by phagolysosomal destruction of the pathogen. **(B)** Under unfavorable growth conditions, *Chlamydia*’s progress through the developmental cycle is blocked and the bacteria can persist inside the viable host cell for prolonged periods of time without inducing host cell death. Under favorable growth conditions in permissive host cells, *Chlamydia* can complete its developmental cycle and form infectious EBs that are released from host cells through either induction of host cell death **(C)** or extrusion **(D)**. During extrusion, host cells remain viable. Complete extrusion can lead to the formation of inclusion-free cells. **(E)** In some instances, host cell mitosis can give rise to one infected and one inclusion-free daughter cell, while in other instances two infected daughter cells can arise.

Infection with *Chlamydia* spp. not always results in the death of the host cell (Fig. 5). For instance, infected cells, in particular macrophages, may clear infection by mediating pathogen degradation in phagolysosomes or via autophagy (Yong, Chi and Kuo 1987; Sun *et al*. 2012a) (Fig. 5A). Host cell death does also not occur during persistent infection (Fig. 5B). The term persistent infection in this context refers to the observation that in cell culture certain unfavorable conditions, such as nutrient deprivation or exposure to penicillin or interferon gamma (IFN-γ), can temporarily interrupt the developmental cycle of *Chlamydia* (Matsumoto and Manire 1970; Beatty, Byrne and Morrison 1993; Coles *et al*. 1993). Under these conditions, the bacteria can survive inside their host cell for extended periods of time without undergoing division, differentiating into EBs, or causing host cell lysis (Matsumoto and Manire 1970; Beatty, Byrne and Morrison 1993; Coles *et al*. 1993; Perfettini *et al*. 2002a; Foschi *et al*. 2020). Persistent bacteria often appear as abnormally enlarged RBs, also known as aberrant bodies (ABs) (Weiss 1950; Beatty, Byrne and Morrison 1993; Coles *et al*. 1993). When conditions improve, persistent bacteria can eventually resume cell division and progression through the development cycle (Matsumoto and Manire 1970; Beatty, Byrne and Morrison 1993; Coles *et al*. 1993), which also includes induction of host cell lysis (Perfettini *et al*. 2002a; Skilton *et al*. 2009; Foschi *et al*. 2020). *In vivo*, this stress program may contribute to long-term persistence of *Chlamydia* in the infected host and to recurrent infections (Beatty, Morrison and Byrne 1994; Schuchardt and Rupp 2016).

Besides bacterial egress involving cell death (Fig. 5C), *Chlamydia* can also be released from host cells by a mechanism that maintains host cell viability (Fig. 5D). Early studies described this process as exocytotic release, extrusion of *Chlamydia* vacuoles or liberation of bacteria in cytoplasmic fragments surrounded by cell membranes (Doughri, Storz and Altera 1972; de la Maza and Peterson 1982; Todd and Caldwell 1985). This extrusion process was studied in greater detail by Hybiske and Stephens (Hybiske and Stephens 2007). Extrusions are bacteria-filled vesicles that pinch off from infected cells. They are bound by membrane derived from the host plasma membrane and contain bacteria encased in an intact inclusion surrounded by a layer of host cell-derived cytoplasm (Hybiske and Stephens 2007). During extrusion, the infected cell may release the entire load of intracellular bacteria or may retain a smaller inclusion (Hybiske and Stephens 2007). While host cells survive the process, it is possible that cells that retain parts of the inclusion will proceed to host cell lysis later. Similarly, released extrusions will eventually lyse to release EBs so that these can infect new host cells. Extrusions also display phosphatidylserine at their surface, which was shown to facilitate uptake of extrusions by professional phagocytes in a manner analogous to the clearance of apoptotic bodies (Zuck *et al*. 2017). While this process enabled enhanced pathogen survival in the phagocytes, likely by preventing phagocyte activation, and was suggested to enable the bacteria to exploit these cells as vehicles for dissemination, it did only infrequently result in productive infection of the phagocytes (Sherrid and Hybiske 2017; Zuck *et al*. 2017). Like the above-mentioned clearance of infection by intracellular degradation of the bacteria, complete extrusion represents another incident in which infected cells can give rise to inclusion-free cells. A third event that can lead to this outcome is the mitotic division of an infected cell, which often gives rise to one uninfected daughter cell (Campbell, Richmond and Yates 1989) (Fig. 5E).

While the majority of the cultured environmental chlamydiae also cause host cell death at the end of their developmental cycle (Kahane *et al*. 1999; Greub and Raoult 2002; Goy, Croxatto and Greub 2008), examples of delayed cell death or long-term co-existence with the host cell exist. For example, in cultures infected with *Simkania negevensis*, the process of bacterial replication and EB formation was completed within ∼3 days of infection, yet release of EBs did not occur before day 12 (Kahane *et al*. 1999; Kahane, Kimmel and Friedman 2002). It is possible that this species lacks a bacterial factor involved in the induction of host cell death. One candidate may be CPAF, as the genome of *S. negevensis* does not encode a CPAF homolog (Collingro *et al*. 2011). Further experimentation is needed to test this idea. In the case of the *Parachlamydiaceae*, which naturally infect free-living amoebae, the growth of the bacteria may be synchronized with the replication of their host (Horn 2008). For instance, depending on the host strain and growth temperature, infection of *Acanthamoeba* spp. with *Protochlamydia amoebophila* or *Parachlamydia acanthamoebae* either resulted in host cell lysis or enabled the establishment of stable co-cultures (Fritsche, Sobek and Gautom 1998; Greub, La Scola and Raoult 2003b).

Overall, the impact of these alternative host cell fates and lifestyles on bacterial replication and survival, host adaptation, pathogenesis and immune responses is only insufficiently understood.

### Replication-independent cytotoxicity of *Chlamydia* spp.

#### Chlamydia toxicity in mice

*Chlamydia* spp. also have a cytotoxic potential that is independent of their ability to establish infection and that has historically been discussed in relation to their *in vivo* toxicity. In the early 1940s, Rake and co-workers demonstrated that intravenous injection of *Chlamydia* (*C. trachomatis* LGV, *C. muridarum* or *C. psittaci*) caused rapid death in mice (Rake and Jones 1943; Rake and Jones 1944). A major proportion of the mice showed signs of toxemia and died within 4–24 h post-inoculation (Rake and Jones 1944). *Chlamydia muridarum* and *C. psittaci*, but not *C. trachomatis*, caused a biphasic curve of death, as some mice that survived the early period after inoculation died up to few weeks later. Yet, in contrast to the deaths that occurred rapidly, these delayed deaths were preceded by typical signs of infection, such as ruffled fur, hunched back and loss of weight (Rake and Jones 1944). Toxicity for mice upon intravenous inoculation was later confirmed by others for various strains of *Chlamydia* (Manire and Meyer 1950; Bell, Snyder and Murray 1959; Wang and Grayston 1963; Taverne, Blyth and Reeve 1964).

The above-mentioned observations led to the idea that *Chlamydia* spp. produce a toxin (Rake and Jones 1944). This hypothetical toxin was suggested to be of low potency, because killing of mice required injection of very high infection doses (Rake and Jones 1944). The 'toxin' could not be separated from the bacteria and any procedure that affected the infectivity of the inoculum, for example formalin treatment or prolonged extracellular incubation, also diminished its toxicity (Rake and Jones 1944). Experiments with purified EBs, RBs and EB cell walls of *C. psittaci* indicated that only the EB form was toxic (Christoffersen and Manire 1969). Moreover, antisera generated against formalin-killed bacteria or during infection with sublethal doses could inactivate the 'toxin' and protect mice from death when they were administered together with toxic doses of intravenously injected bacteria or when they were used to pretreat bacterial suspensions before injection into mice (Rake and Jones 1944; Bell, Snyder and Murray 1959; Wang and Grayston 1963).

#### Immediate cytotoxicity of high multiplicities of infection

In 1976, Moulder and colleagues proposed that the above-described rapid killing of mice can be explained by direct physical damaging of cells resulting from the ingestion of high numbers of bacteria (Moulder *et al*. 1976). The authors showed that high doses of *C. psittaci* caused rapid death of cultured L cells (murine fibroblasts), a phenomenon that was named immediate cytotoxicity (Moulder *et al*. 1976). Indeed, when cells were treated with 500–1000 ID_50_ per cell, where ID_50_ is the dose required to establish infection in 50% of the cells, morphological changes such as rounding were observed as early as 30 min after infection (Moulder *et al*. 1976). Cell monolayers were completely destroyed at 24 h post-infection (hpi) (Moulder *et al*. 1976) and the cells released inorganic ions, an indicator of necrotic death (Chang and Moulder 1978). Immediate cytotoxicity of necrotic nature and variable strength was also observed after infection with high doses of *C. trachomatis* or *C. muridarum*, and in other cell types, such as in HeLa cells and murine macrophages (Moulder *et al*. 1976; Kuo 1978; Wyrick, Brownridge and Ivins 1978).

Chloramphenicol and rifampin, inhibitors of bacterial protein synthesis and transcription, respectively, did not diminish toxicity of high doses of *C. psittaci* for L cells (Moulder *et al*. 1976). Moreover, while UV-inactivated bacteria could invade host cells and cause toxicity, treatments that prevented bacterial entry, such as heat-inactivation of the bacteria or low temperature during inoculation, blocked immediate toxicity of *Chlamydia* (Moulder *et al*. 1976; Kuo 1978). Pretreatment of *C. psittaci* with antisera that had been raised against *C. psittaci* could also block both its infectivity and immediate toxicity (Moulder *et al*. 1976). It thus seemed that immediate cytotoxicity of *C. psittaci*, like its rapid toxicity for mice, was independent of bacterial replication, yet it was dependent on host cell invasion.

When L cells were infected with lower doses of *C. psittaci* (10–100 ID_50_ per cell), both multiplication-independent and multiplication-dependent toxicity were observed, while at doses below 10 ID_50_ per cell, multiplication-independent toxicity disappeared and induction of host cell death was dependent both on entry and intracellular bacterial replication (Kellogg, Horoschak and Moulder 1977; Chang and Moulder 1978). Moreover, at these low doses, host cell damage was only apparent at ∼48–72 hpi, reflecting late stage host cell death (Kellogg, Horoschak and Moulder 1977; Chang and Moulder 1978).

The idea that immediate cytotoxicity was solely a consequence of physical damage caused by ingestion of bacteria was challenged by the finding that different chlamydial species and strains displayed a distinct toxic potential (Belland *et al*. 2001). Moreover, while heat-inactivation of *C. psittaci* abolished its ability to induce immediate toxicity in murine macrophages, the treatment rather enhanced than prevented uptake of the bacteria by these cells and phagocytosis of equivalent numbers of latex beads was non-toxic to the cells (Wyrick, Brownridge and Ivins).

#### Role of LPS in replication-independent toxicity of Chlamydia

Like in other gram negative bacteria, the outer membrane of *Chlamydia* spp. contains a form of LPS (Nurminen *et al*. 1983). LPS is an endotoxin that after release into the bloodstream can cause severe systemic inflammatory reactions, leading to fever, endotoxin shock, tissue injury and death (Galanos and Freudenberg 1993). In this context, LPS mainly acts indirectly through activation of immune cells, which for instance also stimulates secretion of the cytotoxic cytokine TNFα (Galanos and Freudenberg 1993). Chlamydial LPS was shown to possess comparably low endotoxic activity, which was proposed to be attributed to its structural characteristics, such as the higher hydrophobicity of its lipid A moiety (Brade *et al*. 1986; Ingalls *et al*. 1995; Heine *et al*. 2003; Yang *et al*. 2019).

A potential involvement of LPS in *Chlamydia*’s toxicity for mice and in immediate cytotoxicity was studied by Ivins and Wyrick (Ivins and Wyrick 1978). After injection of high doses of *C. psittaci*, similar levels of mortality were observed in the endotoxin-resistant mouse strain C3H/HeJ compared with endotoxin-sensitive C3H/HeN mice. When macrophages derived from these mice were challenged with *C. psittaci*, only a slight reduction in immediate toxicity was observed in cells derived from C3H/HeJ mice (Ivins and Wyrick 1978). The authors further argued that a major role for LPS in *Chlamydia* toxicity appeared unlikely, because LPS is highly heat-stable (Magalhaes *et al*. 2007), whereas the toxic potential of *Chlamydia* was heat-labile (Rake and Jones 1944; Moulder *et al*. 1976; Wyrick, Brownridge and Ivins 1978). A more recent study also showed that intraperitoneal injection of purified *C. trachomatis *E LPS was non-toxic to mice, even when used at 100 times higher amounts than toxic doses of *E.coli* LPS (Yang *et al*. 2019).

Interestingly, *C. trachomatis* LPS was shown to affect the viability of human sperm with 500 times higher potency than *E.coli* LPS (Galdiero *et al*. 1994; Hosseinzadeh, Pacey and Eley 2003). Sperm death occurred rapidly, was accompanied by apoptotic effector caspase activation, and could be partially blocked by Z-VAD-FMK (Eley *et al*. 2005). However, while the toxicity of LPS purified from *C. trachomatis* E or *C. trachomatis* LGV was comparable in strength, purified EBs of *C. trachomatis* E were significantly more toxic than EBs of *C. trachomatis* LGV (Hosseinzadeh, Pacey and Eley 2003). It was thus suggested that also the rapid toxicity for sperm cells could not be explained by LPS alone.

#### Role of bacterial proteins in replication-independent toxicity of Chlamydia

*Chlamydia* spp. produce several proteins that are cytotoxic for human cells upon contact or when ectopically expressed inside the cells. For instance, exposure to the *C. trachomatis* heat shock proteins HSP60 and HSP10 induced rapid death in human fibroblasts and in epithelial cells (Equils *et al*. 2006; Jha *et al*. 2011). This cell death appeared to be of apoptotic nature (Equils *et al*. 2006; Jha *et al*. 2011). *Chlamydia* genomes also encode a protein named *Chlamydia* protein associated with death domains (CADD), which has a domain that is homologous to death domains found in members of the mammalian TNF receptor family (Stenner-Liewen *et al*. 2002). CADD interacted *in vitro* with various death receptors and co-localized with CD95/FAS during infection. However, while ectopic expression of CADD in uninfected cells caused caspase-dependent apoptosis, infected cells were resistant (Stenner-Liewen *et al*. 2002). It is unknown whether the above-mentioned proteins could contribute to the immediate toxicity of *Chlamydia* spp., yet it is unlikely, because immediate cytotoxicity caused by high doses of bacteria appears to be a non-apoptotic form of cell death. Moreover, although the *Chlamydia* protease CPAF induced non-apoptotic cell death when expressed in active form ectopically in the cytosol of uninfected cells (Paschen *et al*. 2008), and may play a role in *Chlamydia* exit (Yang *et al*. 2015), a role in immediate cytotoxicity is less likely, because the protease, or at least the bulk of the protease present in infected cells, was shown to enter the host cell cytosol only at late stages of infection (Snavely *et al*. 2014).

The analysis of the *C. muridarum* genome revealed the presence of three genes (TC0437, TC0438, TC0439) that encode proteins with significant homology to the large cytotoxins A and B of *Clostridium difficile* (Belland *et al*. 2001; Carlson *et al*. 2004). These clostridial toxins act as UDP-glucosyltransferases and interfere with the activity of RHO family GTPases, causing disruption of the actin cytoskeleton, cell rounding and eventually cell death (Carter, Rood and Lyras 2010). The amino acid residues that mediate UDP-glucose binding and glucosyltransferase activity are well conserved in the *C. muridarum* toxin homologs (Belland *et al*. 2001; Carlson *et al*. 2004). Interestingly, the cytotoxin locus differs significantly between *Chlamydia* spp. (Belland *et al*. 2001; Carlson *et al*. 2004). In *C. trachomatis*, only fragmented and/or truncated homologs of TC0438 could be found and homologs of TC0437 or TC0439 were absent. Significant variability could also be seen between distinct strains and potential correlations between toxin genotypes and disease groups were observed (Belland *et al*. 2001; Carlson *et al*. 2004). Genitotropic strains appeared to carry a gene (CT166 in *C. trachomatis* D) that encodes the intact N-terminal part of the toxin, including both the UDP-glucose binding and glycosyltransferase domains. In contrast, ocular strains were found to encode a protein that contains only the UDP-glucose binding domain. Finally, in genomes of LGV strains both domains were absent (Belland *et al*. 2001; Carlson *et al*. 2004).

A possible connection between toxin genes and immediate cytotoxicity was proposed based on the observation that the morphological and cytoskeletal changes induced by high doses of *Chlamydia* were similar than those induced by the clostridial toxins (Belland *et al*. 2001). In addition, a correlation between strength of immediate toxicity and the presence of intact toxin genes was observed (Belland *et al*. 2001). *Chlamydia muridarum* mutants that have nonsense mutations in the toxin genes TC0437 or TC0439 also displayed a slightly reduced immediate toxicity and induced less pathology in a murine genital tract infection model (Rajaram *et al*. 2015). While limited conclusions could be drawn from this study, due to the presence of additional mutations in these strains, these data demonstrated that neither of these toxins alone was sufficient to explain the observed cytotoxicity. Moreover, the fact that LGV strains of *C. trachomatis* typically do not contain an intact toxin gene, but still induce some degree of immediate cytotoxicity (Moulder *et al*. 1976), and cause death in mice (Rake and Jones 1944), suggests that the multiplication-independent toxicity of high doses of *Chlamydia* could be caused by a combination of different factors. It should be noted that the toxin genotype of LGV strains may also be variable. For instance, a recent study reported the isolation of a LGV strain that appeared to be a recombinant between *C. trachomatis* L2 and D strains, carried an intact toxin gene (CT166) and was more cytotoxic towards cultured cells than *C. trachomatis* L2 (Somboonna *et al*. 2011).

An important question that remains is whether the multiplication-independent toxicity of *Chlamydia* is significant for disease manifestation. For instance, it is possible that the products of the *Chlamydia* toxin genes, when present at low amounts, are non-toxic, but rather have specific roles in modulating host cellular processes in favor of bacterial entry, survival and replication. Consistent with this idea, it was shown that CT166 from *C. trachomatis* D acts on the small GTPases RAC, RHOA and RAS and thereby modifies various cellular processes, such as cell proliferation and cell migration (Thalmann *et al*. 2010; Bothe *et al*. 2015). However, cells may encounter high doses of *Chlamydia* when they are exposed to neighboring infected cells or extrusions that lyse and release their entire load of bacteria. In this context, immediate toxicity may contribute to tissue damage and inflammation and potentially pathogen dissemination. The molecular nature of cell death induced by high doses of *Chlamydia* and the potential involvement of host RCD and defense programs also needs to be further explored.

#### Induction of cell death in bystander cells

Apart from multiplication-dependent and -independent effects on the viability of infected cells, *Chlamydia* spp. can also affect the fate of uninfected cells. Dual staining for *Chlamydia* inclusions and hallmarks of apoptosis in epithelial cell cultures infected with various *Chlamydia* species and strains indicated enhanced levels of apoptosis in inclusion-free bystander cells (Ojcius *et al*. 1998; Schöier *et al*. 2001; Greene *et al*. 2004). Augmented levels of apoptosis in inclusion-free cells were also observed in the genital tract of mice infected with *C. muridarum* (Perfettini *et al*. 2000). Furthermore, co-culture with infected macrophages induced apoptosis in uninfected T cells (Jendro *et al*. 2000; Jendro *et al*. 2004; Sessa *et al*. 2009). Several studies suggested that apoptosis in bystander cells is induced by soluble factors, such as TNFα, interferon alpha (IFN-α) and interferon beta (IFN-β), which are secreted by infected cells. For instance, cell-free supernatants of infected macrophage cultures were sufficient to induce apoptosis in T cells and this T cell apoptosis could be blocked by TNFα depletion (Jendro *et al*. 2004; Sessa *et al*. 2009). Moreover, depletion of TNFα in *C. muridarum*-infected mice reduced the incidence of apoptosis in uninfected cells at the site of infection (Perfettini *et al*. 2000). TNFα depletion in mice and guinea pigs also increased the numbers of inflammatory cells in infected tissues, likely by preventing their death (Darville, Andrews and Rank 2000). Similarly, reduced levels of macrophage apoptosis were observed in lung tissues of *C. muridarum*-infected interferon-α/β receptor (IFNAR)-deficient mice compared with wild-type mice (Qiu *et al*. 2008). Besides possible direct contributions to tissue damage, inflammation and post-infection sequelae, *Chlamydia*-induced cell death in bystander cells may therefore also impair anti-chlamydial immune responses via depletion of immune cells.

## ANTI-DEATH ACTIVITIES OF CHLAMYDIAE

### *Chlamydia*-mediated inhibition of apoptosis

#### Premature host cell death disrupts Chlamydia development

**Figure 6.**
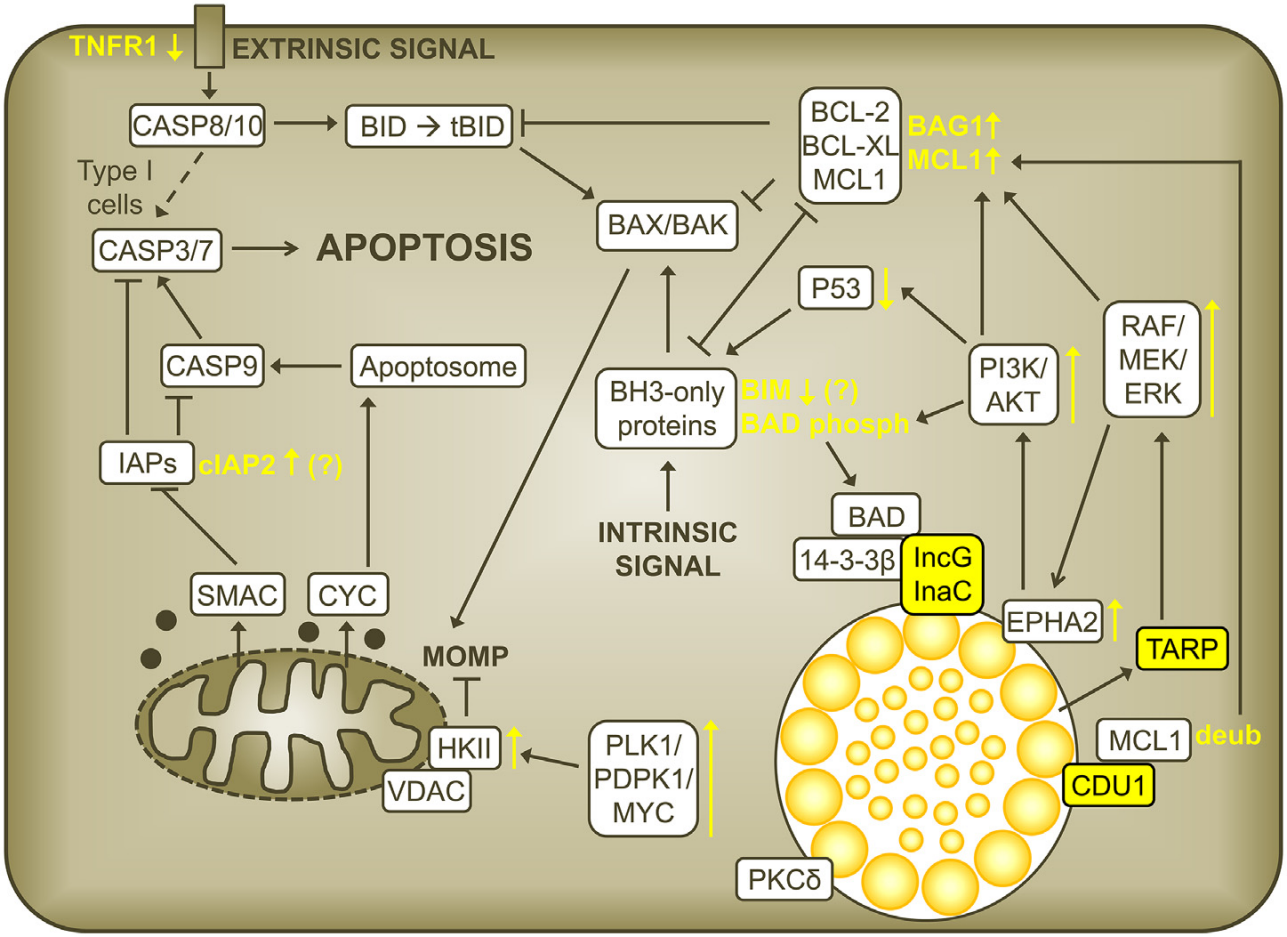
Anti-apoptotic activities of *C. trachomatis*. The illustration gives an overview of the main anti-apoptotic activities described for *C. trachomatis*. *Chlamydia* factors and *Chlamydia*-mediated activities are indicated in yellow. Upward facing arrows next to factors or pathways indicate upregulation or activation; downward facing arrows indicate downregulation. Note that the anti-apoptotic activities of *Chlamydia* spp. were shown to be species specific. For instance, NFκB activation was reported to play a role in apoptosis inhibition during infection with *C. pneumoniae*, but not during infection with *C. trachomatis*. It is therefore not shown.

The reports that late stage host cell death induced by *Chlamydia* spp. proceeds in the absence of caspase activation are in good agreement with the observation that *Chlamydia* spp. inhibit the apoptotic machinery in infected cells (Fig. 6). This phenomenon was first described in 1998 (Fan *et al*. 1998), attracted a lot of attention and was the subject of intensive investigation (Perfettini *et al*. 2003b; Byrne and Ojcius 2004; Häcker, Kirschnek and Fischer 2006; Ying *et al*. 2007; Sharma and Rudel 2009). While newer findings challenged this idea, as discussed below, the anti-apoptotic trait was proposed to enable the pathogen to maintain host cell viability in situations of cellular stress. Premature host cell death can be detrimental for *Chlamydia* spp., because the bacteria depend on a host cell that provides a suitable replicative niche. Moreover, EBs are only formed during the late stage of the developmental cycle and RBs are non-infectious and fragile (Tamura, Matsumoto and Higashi 1967). Consistently, it was shown that experimental induction of apoptosis at mid-stage of infection could abrogate formation of infectious bacteria (Ying *et al*. 2008b). In this system, apoptosis was triggered by inducible overexpression of the pro-apoptotic protein BCL-2-like protein 11 (BIM) in HeLa cells infected with *C. trachomatis* L2 (Ying *et al*. 2008b). While Z-VAD-FMK blocked the morphological signs of apoptosis in these cells, it failed to restore normal production of infectious EBs (Ying *et al*. 2008b). This suggested that even in the absence of caspase-mediated cell demolition, partial induction of the apoptotic pathway, causing MOMP and hence mitochondrial dysfunction, can also impair chlamydial development (Ying *et al*. 2008b).

#### Overview of chlamydial anti-apoptotic activities

Cells infected with *C. trachomatis* or *C. pneumoniae* were shown to be profoundly protected against apoptosis induced by various experimental stimuli. These include conditions that cause intracellular stress and are known to induce the intrinsic pathway of apoptosis in uninfected cells [for instance, UV-irradiation, the DNA-damaging drug etoposide and the kinase inhibitor staurosporine (STS)] (Fan *et al*. 1998; Dean and Powers 2001; Fischer *et al*. 2001; Rajalingam *et al*. 2001; Airenne *et al*. 2002; Fischer *et al*. 2004b; Greene *et al*. 2004). Furthermore, infected cells were also protected against immunological mediators of cell death that activate the extrinsic pathway of apoptosis, such as TNFα and CD95L/FASL (Fan *et al*. 1998; Fischer *et al*. 2001; Rajalingam *et al*. 2001; Airenne *et al*. 2002; Fischer *et al*. 2004a). Resistance to granzyme B/perforin (GRB/PRF)-mediated killing and to poly(I:C)-induced apoptosis was also reported (Fan *et al*. 1998; Böhme *et al*. 2009). Protection against pro-apoptotic stimuli was also observed during infection with other *Chlamydia* spp., including for example *C. psittaci*, *C. caviae* and *C. muridarum*, albeit the degree of protection appeared to vary (Fan *et al*. 1998; Greene *et al*. 2004; Zhong *et al*. 2006; Messinger *et al*. 2015). Furthermore, apoptosis inhibition by *Chlamydia* spp. was observed in a variety of cell lines and primary cells from diverse origins, including epithelial cells, fibroblasts, endothelial cells, monocytes and lymphoid cells (Fan *et al*. 1998; Dean and Powers 2001; Fischer *et al*. 2001; Rajalingam *et al*. 2001; Airenne *et al*. 2002; Fischer *et al*. 2004a,b; Greene *et al*. 2004), and not only during active but also during persistent infection (Dean and Powers 2001; Airenne *et al*. 2002; Paland *et al*. 2006; Li *et al*. 2018).

When *Chlamydia*-infected cells were exposed to the above-mentioned stimuli, they failed to develop typical characteristics of apoptotic cells, such as nuclear condensation and/or fragmentation, DNA double-strand breaks, nucleosomal DNA degradation, proteolytic activation of effector CASP3 and effector caspase activity (Fan *et al*. 1998; Dean and Powers 2001; Fischer *et al*. 2001; Rajalingam *et al*. 2001; Airenne *et al*. 2002; Fischer *et al*. 2004a,b; Greene *et al*. 2004). In infected cultures, protection was restricted to cells carrying considerable inclusions, while inclusion-free cells and cells containing very small inclusions were still susceptible to apoptosis induction (Fan *et al*. 1998; Rajalingam *et al*. 2001; Fischer *et al*. 2004b; Xiao *et al*. 2004; Xiao *et al*. 2005; Zhong *et al*. 2006). When infections were carried out at low multiplicities of infection (MOI), strong resistance to apoptosis-inducing conditions was usually established until ∼24 hpi and was maintained until the end of the infection cycle (Fan *et al*. 1998; Dean and Powers 2001; Rajalingam *et al*. 2001). However, infections at higher MOIs established the same level of resistance earlier (Fan *et al*. 1998; Rajalingam *et al*. 2001). Exposure to heat- or UV-inactivated bacteria failed to mediate apoptosis resistance (Geng *et al*. 2000; Airenne *et al*. 2002; Fischer *et al*. 2004a). Several studies also showed that the anti-apoptotic state could not be established in presence of rifampin or chloramphenicol (Fan *et al*. 1998; Fischer *et al*. 2001; Böhme *et al*. 2009). While some authors interpreted this finding as an indication that apoptosis inhibition depends on bacterial protein synthesis, it should be noted that early addition of these antibiotics would not only inhibit the synthesis of virulence factors, but also entirely prevent the formation of inclusions. Indeed, Fischer et al showed that rifampin was highly effective in blocking *C. pneumoniae*-mediated protection against apoptosis (induced at 72 hpi) when added at the time of infection, but already significantly less effective when added at 6 hpi and ineffective when added at 24 hpi (Fischer *et al*. 2001). Furthermore, chloramphenicol could sensitize cells infected with *C. trachomatis* L2 to poly(I:C)-induced apoptosis (induced at 20 hpi) when added at 2.5 hpi, but not when added at 20 hpi (Böhme *et al*. 2009). LPC-011, which blocks the synthesis of LPS by inhibiting the enzyme UDP-3-O-(R-3-hydroxymyristoyl)-*N*-acetylglucosamine deacetylase (LPXC), also sensitized cells infected with *C. trachomatis* to STS-induced apoptosis (Wang, Rockey and Dolan 2020). Yet the inhibitor is also known to induce abnormal bacterial development and to block inclusion formation when used at high concentrations (Nguyen *et al*. 2011).

#### Inhibition of apoptosis at the pre-mitochondrial and at the post-mitochondrial levels

*Chlamydia* spp. block the apoptosis machinery at various levels. When infected cells were exposed to stimuli that activate the intrinsic pathway, apoptosis was inhibited at a level upstream of MOMP. Activation of BAX and BAK did not occur in infected cells (Fischer *et al*. 2004b; Xiao *et al*. 2004; Paland *et al*. 2006; Zhong *et al*. 2006). Consequently, there was no release of CYC from mitochondria (Fan *et al*. 1998; Dean and Powers 2001; Fischer *et al*. 2001; Rajalingam *et al*. 2001; Airenne *et al*. 2002; Fischer *et al*. 2004a,b; Greene *et al*. 2004; Xiao *et al*. 2004) and no activation of CASP9 (Fischer *et al*. 2001, 2004a; Xiao *et al*. 2005) or CASP3 (Fan *et al*. 1998; Fischer *et al*. 2001; Rajalingam *et al*. 2001; Xiao *et al*. 2004). However, addition of CYC to cell extracts derived from *C. pneumoniae-*infected cells also failed to induce CASP9/3 activation, suggesting that infection also blocked apoptosis at a level downstream of the mitochondria (Fischer *et al*. 2001).

In the context of the extrinsic pathway, most studies suggested that *Chlamydia* spp. fail to block early apoptotic signaling events activated by death receptors (Fischer *et al*. 2004a; Paland *et al*. 2006; Rajalingam *et al*. 2006; Sixt *et al*. 2018). These events include the activation of CASP8 and the cleavage of BID, which connects the extrinsic pathway with the intrinsic pathway. Indeed, *Chlamydia* failed to protect type I cells, in which CASP8 activation is sufficient to activate the apoptotic effector caspases (Kantari and Walczak 2011), from CD95L/FASL-mediated apoptosis (Fischer *et al*. 2004a). However, *Chlamydia* infection could protect type II cells (Fischer *et al*. 2004a), in which a mitochondrial amplification of the apoptotic signal by tBID-mediated activation of BAX/BAK and induction of MOMP is required (Kantari and Walczak 2011). In these cells, exposure to CD95L/FASL (anti-FAS antibody) or TNFα/CHX did not result in BAX/BAK activation, release of mitochondrial CYC or activation of CASP9/3 (Fan *et al*. 1998; Rajalingam *et al*. 2001; Fischer *et al*. 2004a; Xiao *et al*. 2005; Paland *et al*. 2006).

#### Induction of survival signaling pathways

*Chlamydia trachomatis* activates several survival signaling pathways in infected cells. These include for instance the phosphoinositide 3-kinase/protein kinase B (PI3K/AKT) pathway and the RAF/MEK/ERK mitogen-activated protein kinase (MAPK) pathway (Verbeke *et al*. 2006; Paland *et al*. 2008; Rajalingam *et al*. 2008; Du *et al*. 2011; Kun *et al*. 2013; Siegl *et al*. 2014). Importantly, depletion of AKT using specific siRNAs or pharmacologic inhibition of PI3K with LY294002 sensitized infected cells to STS- and GRB-induced apoptosis (Verbeke *et al*. 2006; Rajalingam *et al*. 2008). In the presence of LY294002, STS also induced the release of mitochondrial CYC in infected cells, suggesting that the induction of the PI3K/AKT pathway contributes to the apoptotic block at the pre-mitochondrial level (Verbeke *et al*. 2006). The MEK inhibitor U0126 and the RAF inhibitor GW5074 also sensitized infected cells to STS- and GRB- induced apoptosis (Rajalingam *et al*. 2008; Du *et al*. 2011; Kun *et al*. 2013). Moreover, *C. trachomatis* L2 also induces the polo-like kinase 1/3-phosphoinositide-dependent protein kinase 1/Myc proto-oncogene (PLK1/PDPK1/MYC) signaling pathway, which was shown to contribute to protection against TNFα/CHX-induced apoptosis (Al-Zeer *et al*. 2017).

*Chlamydia trachomatis* induces survival pathways by various means. For instance, a recent study indicated that the host surface receptor Ephrin A2 (EPHA2) acts as receptor for *Chlamydia* EBs during the early stage of infection, but is subsequently internalized, remains associated with the *Chlamydia* inclusion and mediates lasting PI3K activation (Subbarayal *et al*. 2015). The ERK pathway was involved in infection-induced upregulation of EPHA2 and infection-induced AKT activation was found to depend on EPHA2 signaling during mid-phase of infection (Subbarayal *et al*. 2015). Depletion of EPHA2 sensitized cells to TNFα/CHX-induced apoptosis at 16 hpi (Subbarayal *et al*. 2015). Moreover, one study linked early ERK activation to the secreted *Chlamydia* effector protein translocated actin recruiting phosphoprotein (TARP), which phosphorylates Src homology 2 domain-containing-transforming protein C1 (SHC1), which in turn activates the MEK/ERK pathway (Mehlitz *et al*. 2010). Depletion of SHC1 blocked early ERK activation and sensitized *C. trachomatis*-infected cells to TNFα/CHX-induced apoptosis at 6 hpi (Mehlitz *et al*. 2010). However, it should be noted that a very high MOI of 50 had to be used in this experiment to establish protection against apoptosis in control cells at this early time point during infection. Recent studies also suggested that the plasmid-encoded secreted *Chlamydia* protein PGP3 may contribute to apoptosis inhibition (He *et al*. 2019), likely via induction of the ERK signaling pathway (Luo *et al*. 2019).

During infection with other *Chlamydia* spp., alternative signaling pathways have been implicated in promoting host cell survival. For instance, PI3K inhibition did not sensitize *C. pneumoniae*-infected HeLa cells to STS-induced apoptosis (Verbeke *et al*. 2006). Instead, infection with *C. pneumoniae* induced nuclear factor kappa B (NFκB) activation in human epithelial cells (Paland *et al*. 2006). CAPE, an inhibitor of NFκB nuclear translocation, as well as siRNA-mediated depletion of the P65 subunit of NFκB, sensitized infected cells to TNFα/CHX- and STS-induced apoptosis (Paland *et al*. 2006). *Chlamydia pneumoniae* also induced NFκB activation in the human monocytic cell line Mono Mac 6 (Wahl *et al*. 2001, 2003). Because inhibition of NFκB activation induced apoptosis in these cells, the authors suggested that NFκB activation during infection was important for maintenance of host cell survival (Wahl *et al*. 2001, 2003). In contrast to these findings, no activation of NFκB, assessed by monitoring of NFκB nuclear translocation and IκB degradation, was observed in human epithelial cells infected with *C. trachomatis* (Xiao *et al*. 2005). Furthermore, inhibition of NFκB activation in these cells did not result in sensitization to STS- or TNFα/CHX-induced apoptosis, and P65-deficient *C. trachomatis*-infected MEFs maintained protection against TNFα/CHX-induced apoptosis (Xiao *et al*. 2005). In the case of *C. psittaci*, little is known about the contribution of survival pathways to anti-apoptosis. Yet, induction of the janus kinase /signal transducer and activator of transcription protein 3 (JAK/STAT3) pathway was proposed to contribute to apoptosis resistance during infection with this species (Sun *et al*. 2017). Moreover, persistent infection of HeLa cells with *C. psittaci* also activated the ERK pathway and U0126 sensitized infected cells to STS-induced apoptosis (Li *et al*. 2018).

It should be mentioned that the use of pharmacologic inhibitors for the testing of the contribution of specific signaling pathways to *Chlamydia* anti-apoptosis is complicated by the fact that certain inhibitors, at least at high concentrations and when added early during infection, affect chlamydial growth. Because cells that contain only small inclusions are not well protected from apoptosis (Rajalingam *et al*. 2001; Zhong *et al*. 2006), it is plausible that each condition that blocks inclusion establishment or growth may also interfere with *Chlamydia* anti-apoptosis, independently of whether the drug target is involved in apoptosis inhibition or not. While some authors included respective controls, this issue was not accounted for in all studies.

#### Interference with pro-apoptotic BCL-2 family proteins

Various explanations for the profound *Chlamydia*-mediated block of the apoptotic machinery upstream of MOMP have been proposed. The majority of these suggest that *Chlamydia* interferes with the balance between pro-apoptotic BH3-only proteins and anti-apoptotic BCL-2 family proteins, which controls the activation state of BAX and BAK and thus regulates MOMP. Initially, based on the analysis of host protein levels in lysates of infected cells, it was proposed that *Chlamydia* spp. cause the degradation of BH3-only proteins (Fischer *et al*. 2004b; Dong *et al*. 2005; Ying *et al*. 2005). Degradation of BIM appeared to start at ∼14–16 hpi and to be complete at ∼24–26 hpi (Fischer *et al*. 2004b), a time point that correlated well with the time at which potent protection against apoptosis was established in infected cells. Similar observations were made for other BH3-only proteins, including BCL-2-binding component 3 (PUMA), BCL-2-modifying factor (BMF), Phorbol-12-myristate-13-acetate-induced protein 1 (NOXA), BCL-2-interacting killer (BIK) and tBID (Fischer *et al*. 2004b; Dong *et al*. 2005; Ying *et al*. 2005). BCL-2-associated agonist of cell death (BAD) was reported to be degraded in some studies (Fischer *et al*. 2004b; Ying *et al*. 2005; Verbeke *et al*. 2006), but not in others (Dong *et al*. 2005). The loss of BH3-only proteins was explained by proteolytic activity, because it could be prevented by addition of proteasome inhibitors and because mRNA levels appeared to be unaffected (Fischer *et al*. 2004b; Ying *et al*. 2005). However, other authors reported that the levels of BH3-only proteins remained unchanged during infection (Rajalingam *et al*. 2008). Today, it is clear that the occasionally observed degradation was caused by the *Chlamydia* protease CPAF, yet only post-lysis in sample buffer when conditions used for the generation of protein samples were inappropriate for blocking residual CPAF activity (Chen *et al*. 2012).

While the broad degradation of BH3-only proteins was revealed as an experimental artifact, there is still a debate concerning BIM, which one group reported to be degraded even when samples were prepared in a way that prevented CPAF post-lysis activity (Dille *et al*. 2015). Furthermore, *Chlamydia* infection was also proposed to interfere with BH3-only proteins in other ways. For instance, infection with *C. trachomatis* L2 caused phosphorylation of BAD, which promotes its association with the host protein 14-3-3β, which in turn was recruited to the *Chlamydia* inclusion (Verbeke *et al*. 2006). Significantly, inhibition of PI3K with LY294002, which sensitized cells to STS-induced apoptosis, blocked BAD phosphorylation and recruitment (Verbeke *et al*. 2006). The authors proposed that BAD sequestration at the inclusion interferes with its pro-apoptotic function. Recruitment of 14-3-3β was proposed to be mediated by the Inc protein IncG (Scidmore and Hackstadt 2001). Consistent with this idea, *C. pneumoniae*, a species that lacks an IncG homolog, did not recruit 14-3-3β (Scidmore and Hackstadt 2001) and protected infected cells from STS-induced apoptosis in a PI3K-independent manner (Verbeke *et al*. 2006). However, genetic evidence from experiments with a *C. trachomatis* IncG mutant still awaits to be collected. It is possible that other bacterial factors could also be involved. For instance, reduced recruitment of 14-3-3β to inclusions was observed during infection with a *C. trachomatis* mutant that was deficient for an Inc protein named inclusion membrane protein for actin assembly (InaC) (Kokes *et al*. 2015).

#### Interference with anti-apoptotic BCL-2 family proteins

Infection with *C. trachomatis* L2 did not affect the expression or stability of the anti-apoptotic BCL-2 family proteins BCL-2 or B-cell lymphoma-extra-large (BCL-XL) (Dong *et al*. 2005; Ying *et al*. 2005). However, levels of induced myeloid leukemia cell differentiation protein (MCL1), another anti-apoptotic member of the BCL-2 family, were strongly upregulated in infected human epithelial cells, at both the mRNA and the protein level (Hess *et al*. 2001; Rajalingam *et al*. 2008). The MEK inhibitor U0126 reduced MCL1 mRNA and protein levels and the PI3K inhibitor LY294002 reduced MCL1 protein levels, suggesting a RAF/MEK/ERK-pathway-dependent transcriptional upregulation of MCL1 and a PI3K-dependent stabilization of MCL1 protein levels in infected cells (Rajalingam *et al*. 2008). Stabilization of MCL1 in *C. trachomatis*-infected cells was also shown to be a consequence of reduced MCL1 ubiquitination and hence reduced proteosomal degradation (Fischer *et al*. 2017). This appeared to be mediated by the *Chlamydia* deubiquitinating enzyme 1 (CDU1), which localizes to the inclusion membrane (Fischer *et al*. 2017). Co-immunoprecipitation experiments and *in vitro* binding assays indicated that CDU1 and MCL1 directly interact with each other and an *in vitro* deubiquitination assay confirmed that MCL1 is a substrate for CDU1 (Fischer *et al*. 2017). While MCL1 levels were reduced during infection with a CDU1-deficient mutant of *C. trachomatis*, compared with infection with wild-type bacteria, MCL1 levels were still increased compared with uninfected cells and cells infected with the mutant were not sensitized to TNFα/CHX-induced apoptosis (Fischer *et al*. 2017). This finding can likely be explained by *Chlamydia* using redundant mechanisms to maintain elevated MCL1 levels. Indeed, depletion of MCL1 using siRNAs or shRNAs could sensitize *C. trachomatis*-infected cells to apoptosis induced by STS, TNFα/CHX or GRB (Rajalingam *et al*. 2008), at least at the mid-stage of infection (24 hpi). No sensitization was seen at later stages of infection (48 hpi) or in cells containing large inclusions (Rajalingam *et al*. 2008). Contrasting with the results reported for human cells, in MEFs, MCL1 deficiency only caused a mild sensitization to STS-induced apoptosis (Ying *et al*. 2008a). However, enhanced levels of MCL1 were also seen in HeLa cells that were persistently infected with *C. psittaci* and in human neutrophils that were infected with *C. pneumoniae* (Sarkar *et al*. 2015; Li *et al*. 2018).

Besides MCL1, other anti-apoptotic proteins may be upregulated during infection and may contribute to maintenance of host cell survival. For example, one study showed that *C. trachomatis* (and *C. muridarum*) infection increased the levels of BCL-2-associated athanogene 1 (BAG1), a BCL-2 binding protein, in a manner dependent on the RAF/MEK/ERK signaling pathway (Kun *et al*. 2013). Depletion of BAG1 sensitized infected cells to STS- and TNFα/CHX-induced apoptosis (Kun *et al*. 2013). An importance of *de novo* host protein synthesis for *Chlamydia* anti-apoptosis appears to disagree with the observation that CHX did not generally sensitize infected cells to apoptosis (Fan *et al*. 1998; Airenne *et al*. 2002; Fischer *et al*. 2004b). It is possible that *Chlamydia* infection primarily affects levels of anti-apoptotic proteins by protein stabilization, rather than by modulating their expression, or that redundant anti-apoptotic strategies can compensate for effects mediated by CHX.

#### Inhibition of apoptosis at a post-mitochondrial stage by IAPs

The inhibitors of apoptosis proteins (IAPs) constitute a group of proteins that have a baculovirus IAP repeat (BIR) domain that allows for direct interaction with caspases (Salvesen and Duckett 2002). IAPs regulate caspase activity, for instance by mediating caspase ubiquitination, which promotes proteosomal degradation (Vaux and Silke 2005). During MOMP, the IAP-inhibiting protein second mitochondria-derived activator of caspases (SMAC) is released from mitochondria together with CYC to promote apoptosis (Du *et al*. 2000; Verhagen *et al*. 2000). A role for IAPs in *Chlamydia*-mediated apoptosis inhibition may therefore depend on *Chlamydia*’s simultaneous capacity to prevent or reduce MOMP. Moreover, conflicting reports on the involvement of IAPs in *Chlamydia* anti-apoptosis have been published. Infection of human epithelial cells with *C. trachomatis* L2 was reported to cause transcriptional upregulation of cellular inhibitor of apoptosis protein 2 (cIAP2), but not cellular inhibitor of apoptosis protein 1 (cIAP1) or X-linked inhibitor of apoptosis (XIAP) (Rajalingam *et al*. 2006). In this study, depletion of cIAP2 using specific siRNAs sensitized infected cells, except such that contained very large inclusions, to TNFα/CHX-induced apoptosis (Rajalingam *et al*. 2006). Interestingly, sensitization was also observed in cells depleted for cIAP1 or XIAP, an observation that the authors explained with the finding that IAPs act in complexes and can affect the stability of each other (Rajalingam *et al*. 2006). However, another study reported that neither depletion of IAPs nor addition of SMAC mimetics (IAP inhibitors) sensitized *C. trachomatis*-infected HeLa cells to TNFα/CHX-induced apoptosis (Waguia Kontchou *et al*. 2016). Moreover, deficiency for XIAP, cIAP1, cIAP2, or cIAP1 and cIAP2 did not sensitize *C. trachomatis*-infected MEFs to TNFα/CHX-induced apoptosis (Ying *et al*. 2008a). In contrast, enhanced levels of cIAP2 mRNA and protein were also observed in human cells infected with *C. pneumoniae* (Wahl *et al*. 2003; Paland *et al*. 2006) and depletion of cIAP2 or cIAP1 sensitized these cells to TNFα/CHX- and STS-induced apoptosis (Paland *et al*. 2006). In these cells, upregulation of cIAP2 could be blocked by CAPE, an inhibitor of NFκB nuclear translocation, and MG132, a proteasome inhibitor that blocks IκB degradation (Wahl *et al*. 2003; Paland *et al*. 2006). It was therefore proposed to be NFκB-dependent in the context of *C. pneumoniae* infection.

#### Interference with CASP8 activation

Two studies reported conflicting observations in respect to direct effects of *C. trachomatis* L2 infection on TNFR1 signaling. One study reported that infection caused a reduction of surface-exposed TNFR1 (Paland *et al*. 2008). Interestingly, total TNFR1 expression was increased in infected cells, yet more TNFR1 was shed into culture supernatants or was internalized (Paland *et al*. 2008). These changes were dependent on the induction of the RAF/MEK/ERK signaling pathway (Paland *et al*. 2008). In contrast, another study showed that *C. trachomatis* blocked TNFR1 internalization, an event that is required for TNFR1-mediated pro-apoptotic signaling (Waguia Kontchou *et al*. 2016). Consistent with this idea, but in conflict with reports from other groups (Paland *et al*. 2006; Rajalingam *et al*. 2006; Sixt *et al*. 2018), the authors found that infection blocked TNFα/CHX-induced processing of CASP8 and BID (Waguia Kontchou *et al*. 2016). A role for cellular FLICE-like inhibitory protein (cFLIP), a regulatory protein that can prevent recruitment and activation of CASP8 (Irmler *et al*. 1997), in apoptosis inhibition was excluded in this context, because cFLIP depletion did not sensitize infected cells to TNFα/CHX-induced apoptosis (Waguia Kontchou *et al*. 2016). In contrast, cFLIP was implicated in *C. trachomatis*’ ability to protect HeLa cells from poly(I:C)-induced apoptosis (Böhme *et al*. 2009). In this system, infection blocked poly(I:C)-induced activation of CASP8, CASP9 and CASP3, as well as BID cleavage (Böhme *et al*. 2009). While cFLIP protein levels were not affected by infection, depletion of cFLIP sensitized infected cells to poly(I:C)-induced CASP8 activation (Böhme *et al*. 2009).

#### Anti-apoptotic (side) effects of metabolic reprogramming

*Chlamydia* spp. modulate host cell metabolism to their benefit and do so in part by mechanisms that can modify host cell resistance to apoptosis. For instance, activation of MYC in *C. trachomatis*-infected cells via the PLK1/PDPK1/MYC signaling pathway was shown to result in upregulation of hexokinase-II (HKII) and enhanced association of HKII with the mitochondrial voltage-dependent anion channel (VDAC) (Al-Zeer *et al*. 2017). While this can promote glycolysis and oxidative phosphorylation, HKII can thereby also compete with pro-apoptotic signals that could otherwise act on VDAC (Pastorino, Shulga and Hoek 2002; Majewski *et al*. 2004). Indeed, dissociation of HKII from VDAC using clotrimazole or N-HKII (a competitive peptide), sensitized *C. trachomatis*-infected cells to TNFα/CHX-induced apoptosis (Al-Zeer *et al*. 2017).

*Chlamydia trachomatis* infection was also shown to cause a pronounced reduction in tumor suppressor P53 protein levels in infected cells (Gonzalez *et al*. 2014; Siegl *et al*. 2014). This seems to be the result of several redundant mechanisms. For instance, infection induced PI3K/AKT-dependent activation of the E3 ubiquitin-protein ligase MDM2 (also called HDM2 in human cells), a protein that mediates ubiquitination and proteosomal degradation of P53 (Gonzalez *et al*. 2014; Siegl *et al*. 2014). Indeed, LY294002, proteasome inhibitors, inhibitors of P53-MDM2 interaction (nutlin3a and RITA), and depletion of MDM2 rescued P53 levels in infected cells (Gonzalez *et al*. 2014; Siegl *et al*. 2014). Another study reported that *C. trachomatis* induced upregulation of miR-30c-5p, a miRNA that is known to downregulate expression of P53 (Chowdhury *et al*. 2017). Interference with miR-30c-5p function resulted in enhanced levels of P53 in infected cells (Chowdhury *et al*. 2017). Infection with other *Chlamydia* spp., such as *C. pneumoniae*, *C. psittaci* and *C. muridarum*, also triggered a drop in P53 levels (Gonzalez *et al*. 2014; Siegl *et al*. 2014). However, while a reduction in P53 levels was observed in various human cells, including HeLa cells, HUVECs and primary cells from fallopian tubes, it was not observed in MEFs (Gonzalez *et al*. 2014; Siegl *et al*. 2014). Importantly, P53 is a pro-apoptotic protein and nutlin3a partially re-sensitized *C. trachomatis*-infected HeLa cells to TNFα/CHX-induced apoptosis (Gonzalez *et al*. 2014). However, experimental interference with P53 downregulation by itself did not induce massive apoptosis in infected cells, but disrupted *Chlamydia* development (Gonzalez *et al*. 2014; Siegl *et al*. 2014). Reduced P53 levels may therefore benefit *Chlamydia* spp. primarily by contributing to the metabolic reprogramming of the host cell (Siegl *et al*. 2014).

Another study suggested that *C. trachomatis* interferes with the pro-apoptotic function of protein kinase C delta (PKCδ) by sequestering the kinase at the *Chlamydia* inclusion (Tse *et al*. 2005). Expression of a PKCδ variant that is catalytically active, but that lacks its diacylglycerol binding domain and can thus not be recruited to the inclusion membrane, induced apoptosis in infected cells (Tse *et al*. 2005). However, in uninfected cells, PKCδ typically localizes to the Golgi apparatus and the expression of the mutant variant that failed to bind lipids induced apoptosis in these cells as well (Tse *et al*. 2005). It is possible that the sequestration of PKCδ at the *Chlamydia* inclusion reflects the acquisition of Golgi-derived host lipids by the *Chlamydia* inclusion, rather than a *bona fide* anti-apoptotic virulence strategy.

#### Inhibition of apoptosis by environmental chlamydiae

The anti-apoptotic capacities of environmental chlamydiae are not well explored, yet available literature indicates species-specific differences. The *Parachlamydiaceae* may lack anti-apoptotic activities and appear to induce apoptosis instead (Greub, Mege and Raoult 2003a; Ito *et al*. 2012; Sixt *et al*. 2012; Matsuo *et al*. 2013; Brokatzky, Kretz and Häcker 2020), as discussed in more detail below. A single study reported that *S. negevensis* could protect HeLa cells from STS- and TNFα/CHX-induced apoptosis (Karunakaran, Mehlitz and Rudel 2011). Apoptotic signaling was blocked at a pre-mitochondrial step. Indeed, infection prevented activation of BAX and BAK, release of mitochondrial CYC, and activation of CASP9 and CASP3, while CASP8 was still activated in presence of TNFα/CHX (Karunakaran, Mehlitz and Rudel 2011). The authors did not observe changes in the levels of the BH3-only proteins or the anti-apoptotic proteins BCL-2 and MCL1, yet cIAP1 and cIAP2 were upregulated in infected cells (Karunakaran, Mehlitz and Rudel 2011). Furthermore, depletion of cIAP1 and/or cIAP2, as well as inhibition of PI3K sensitized infected cells to TNFα/CHX-induced apoptosis (Karunakaran, Mehlitz and Rudel 2011). *Rhabdochlamydia porcellionis*, a species that naturally infects arthropod hosts, was shown to inhibit actinomycin D- and UV-induced apoptosis in infected insect cells (Sixt *et al*. 2013). In contrast, *Waddlia chondrophila*, a species that has been isolated from aborted bovine fetuses, failed to block STS-induced apoptosis in HeLa cells (Dille *et al*. 2015).

#### Significance of Chlamydia-mediated apoptosis inhibition

The profound inhibition of the apoptotic machinery in *Chlamydia*-infected cells has commonly been explained as an attempt of the bacteria to protect host cells from infection-induced stress and to maintain host cell viability under adverse growth conditions or when the cell is under attack by immune mediators (Ying *et al*. 2007; Sharma and Rudel 2009). Indeed, a recent study described that in spite of *C. trachomatis*’ anti-apoptotic trait, low levels of MOMP (known as minority MOMP), were induced in infected cells in a BAX/BAK-dependent manner (Brokatzky *et al*. 2019). The finding was interpreted as evidence for the presence of pro-apoptotic signals in infected cells, explaining the need for potent bacteria-mediated suppression (Brokatzky *et al*. 2019).

However, while interference with *Chlamydia*’s anti-apoptotic strategies could re-sensitize host cells to pro-apoptotic conditions, as described above, it did not cause massive spontaneous cell death in infected cells. Moreover, a recent study in which my co-workers and I monitored the effect of pro-apoptotic conditions on the fate of human epithelial cells infected with *C. trachomatis* L2 by live cell imaging demonstrated that infected cells were not protected from cell death (Sixt *et al*. 2018). While apoptosis inducers, such as STS and TNFα/CHX, failed to induce typical hallmarks of apoptosis in infected cells, these cells died by a necrotic type of death accompanied by early loss of plasma membrane integrity. This necrotic death was also not significantly delayed compared with apoptotic death induced by the same conditions in uninfected cells (Sixt *et al*. 2018). Mechanistically, TNFα/CHX-induced necrosis of infected cells was independent of the necroptotic signaling pathway, but was dependent on CASP8, whose activation was not blocked by infection (Sixt *et al*. 2018). Consistent with the inability of *C. trachomatis* to protect its growth niche under the tested pro-apoptotic conditions, pro-apoptotic stimulation abolished formation of infectious progeny. Yet, in the presence of TNFα/CHX, progeny formation could be rescued by inhibition of CASP8 or CASP8 deficiency (Sixt *et al*. 2018).

While it may seem at first glance puzzling that these observations have not been reported before, it is plausible that the phenomenon has been overlooked, because previous studies primarily focused on the detection of specific hallmarks of apoptosis and used very short periods of incubation with apoptosis-inducing conditions. Moreover, consistent with the findings of our recent study (Sixt *et al*. 2018), Jungas *et al*. reported that STS failed to induce apoptosis in *Chlamydia*-infected cells, but still caused a reduction in cell numbers in cultures of infected adherent cells (Jungas *et al*. 2004). Further experimentation will be required to test whether similar observations can be made during infection with other *Chlamydia* spp. and in primary cells. Moreover, a better understanding of the nature of the pro-apoptotic signals that arise during infection is required.

It may also seem counterintuitive that *Chlamydia* spp. use so many redundant mechanisms to block apoptosis, if these strategies are not sufficient to maintain cell viability. However, it is possible that *Chlamydia* anti-apoptosis, resulting in preferential host cell death by necrosis, is either primarily a side effect of the metabolic reprogramming of the host cell, or that it has other benefits for the bacteria *in vivo*. For instance, apoptotic death of infected cells, in contrast to necrotic death, could be expected to lead to preferential uptake by phagocytes, which are not the preferred host cells for *Chlamydia*, have a high capacity to restrict *Chlamydia* growth, and can stimulate immune responses for example by antigen (cross)-presentation. Moreover, while the uptake of bacteria encased within apoptotic bodies may forestall phagocyte activation, it may reduce the bacteria's capacity to interact with their new host cell and to modulate its biology in a similar way as free bacteria could do. In the future, it will therefore be important to clarify in which ways distinct modes of host cell death can affect survival and spread of the bacteria, disease progression and the immune response.

#### Chlamydia-mediated prolongation of the life span of short-lived cells

In contrast to the above-mentioned observations in *C. trachomatis*-infected epithelial cells, in which *Chlamydia*-mediated inhibition of apoptosis did not achieve prolonged maintenance of host cell viability (Sixt *et al*. 2018), it is well established that infection with *C. pneumoniae* can prolong the life span of certain inherently short-lived cells, such as neutrophils, by delaying apoptotic death (van Zandbergen *et al*. 2004; Rupp *et al*. 2009; Sarkar *et al*. 2015). The mechanism behind this pro-survival effect in neutrophils appears to be distinct from *Chlamydia*-mediated anti-apoptosis in epithelial cells, as it may at least in part represent a non-specific response of neutrophils to infection conditions. Indeed, exposure to *Chlamydia* LPS, heat-killed bacteria or recombinant interleukin 8 (IL-8), a cytokine secreted by infected cells, also enhanced the life span of neutrophils (van Zandbergen *et al*. 2004; Sarkar *et al*. 2015). It was also shown that *C. pneumoniae* caused a PI3K-dependent activation of NFκB in infected neutrophils (Sarkar *et al*. 2015). Inhibitors of PI3K or NFκB activation could reduce infection-induced IL-8 release and could partially re-sensitize infected neutrophils to spontaneous apoptosis (Sarkar *et al*. 2015). The MEK inhibitor U0126 could also re-sensitize infected neutrophils to spontaneous apoptosis (Sarkar *et al*. 2015).

Importantly, the above-mentioned studies showed that apoptosis induction in *C. pneumoniae*-infected neutrophils was only delayed, not entirely blocked. Indeed, it was suggested that apoptotic neutrophils may serve as Trojan Horses enabling the bacteria to be taken up into macrophages in a manner that forestalls macrophage activation and allows prolonged intracellular bacterial survival in the phagocyte, which could then serve as vehicle for pathogen dissemination (Rupp *et al*. 2009). As discussed above, how infection can be established in a macrophage after uptake of bacteria entrapped within a host cell corpse, and if this is a species-specific trait, needs to be further explored.

### *Chlamydia*-mediated inhibition of defensive host cell death

#### Host cell death as a cell-autonomous defense response

Cell-autonomous immunity is the capacity of eukaryotic cells, including non-immune cells of metazoan origin, to detect invading pathogens and to respond to the threat by inducing cellular defense responses (Randow, MacMicking and James 2013). Host cell death is one of these responses, because the controlled demise of infected cells can limit the replication and spread of intracellular pathogens (Jorgensen, Rayamajhi and Miao 2017). As will be discussed below, current knowledge suggests that *Chlamydia* spp. can trigger several cell death-inducing defense responses that have the potential to effectively disrupt *Chlamydia* development and replication. However, the bacteria also have evolved powerful counterstrategies, most notable such that shield the bacteria from the host cell's innate immune sensors.

#### Apoptosis restricts growth of Parachlamydiaceae in animal cells

In certain host-pathogen systems, apoptosis is induced as a defense mechanism that can block pathogen replication, if not actively counteracted by the pathogen. This has been well illustrated by Clem and co-workers, who demonstrated the importance of anti-apoptotic virulence factors for the growth of baculovirus in insect cells (Clem and Miller 1993; Clarke and Clem 2003). Studies with environmental chlamydiae, in particular studies with the naturally amoeba-infecting *Parachlamydiaceae*, suggested that apoptosis might have a similar protective potential against members of the phylum *Chlamydiae*. Certain animal-derived cells, including insect cells and human cells (epithelial cells and macrophages), were shown to induce rapid (or at least premature) cell death in response to infection with *P. amoebophila* and/or *Pa. acanthamoebae* (Greub, Mege and Raoult 2003a; Ito *et al*. 2012; Sixt *et al*. 2012; Matsuo *et al*. 2013; Brokatzky, Kretz and Häcker 2020). This cell death was confirmed to be of apoptotic nature, as it was accompanied by typical apoptotic changes in nuclear morphology, nucleosomal DNA fragmentation, DNA double-strand breaks, externalization of phosphatidylserine and activation of apoptotic effector caspases (Greub, Mege and Raoult 2003a; Ito *et al*. 2012; Sixt *et al*. 2012; Matsuo *et al*. 2013; Brokatzky, Kretz and Häcker 2020). Induction of apoptosis was dependent on the viability of the bacteria and required direct contact between the bacteria and the host cells (Ito *et al*. 2012; Sixt *et al*. 2012; Brokatzky, Kretz and Häcker 2020). Host cell apoptosis was blocked in presence of the pan caspase inhibitor Z-VAD-FMK (Ito *et al*. 2012; Sixt *et al*. 2012; Matsuo *et al*. 2013). BAX/BAK-deficiency or overexpression of BCL-XL could diminish cell death in infected HeLa cells (Brokatzky, Kretz and Häcker 2020). Interestingly, in the insect cell model, inhibition of apoptosis during infection with *Pa. acanthamoebae* was sufficient to maintain host cell viability and to enable effective replication and formation of infectious EBs (Sixt *et al*. 2012). Beneficial effects of apoptosis inhibition on *Pa. acanthamoebae* survival in human host cells were also described more recently (Brokatzky, Kretz and Häcker 2020). It is currently unknown how host cell death is induced during infection with *Parachlamydiaceae*. Moreover, cell death was not observed in each host/pathogen system studied. For instance, apoptosis induction was not observed in primary peripheral blood mononuclear cells (PBMCs) that were infected with *P. amoebophila* or in HEp2 cells that were infected with *Pa. acanthamoebae* (Ito *et al*. 2012).

#### *Chlamydia* spp. actively counteract premature host cell death

The findings discussed above could in principle be explained by an inability of *Parachlamydiaceae* to protect their host cells against apoptosis caused by infection-induced cellular stress, due to a lack of anti-apoptotic virulence strategies. However, given the considerations described above, which challenge the existence of such strong pro-death signals and the potency of *Chlamydia*’s anti-apoptotic trait, it is also plausible that cell death induced during infection with *Parachlamydiaceae* is a host defense response. That means more critical than the bacteria's inability to target the cell death machineries per se, may be an inability to block the defense pathways that act upstream of their induction.

**Figure 7.**
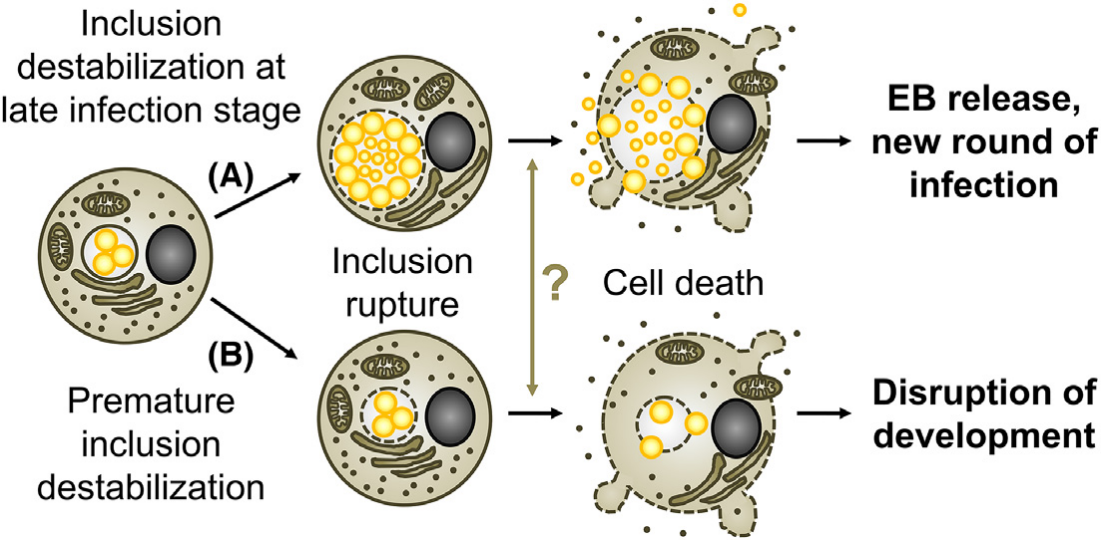
Host cell death as consequence of inclusion instability. **(A)** Host cell lysis at late stages of infection with *C. trachomatis* is preceded by inclusion rupture. **(B)** Conditions that cause premature inclusion rupture, such as laser-mediated disruption of inclusions and potentially lack of certain Inc proteins, result in premature host cell death. The molecular machineries that execute cell death in these distinct situations are not well understood. Yet, it is conceivable that *Chlamydia* co-opts pre-existing host cellular defense responses to mediate its exit from its host cell.

Indeed, recent studies that exploited new genetic tools for *Chlamydia* spp. demonstrated that *C. trachomatis* actively counteracts cellular defense pathways that activate premature host cell death (Sixt *et al*. 2017; Weber *et al*. 2017). More precisely, it was shown that *C. trachomatis* mutants that were deficient for certain Incs, such as *Chlamydia* promoter of survival (CpoS)/CT229, CT383 or IncC/CT233, induced premature host cell death, which impeded formation of infectious EBs (Sixt *et al*. 2017; Weber *et al*. 2017). This host cell death was at least partially dependent on the intracellular innate immune sensor Stimulator of Interferon Genes (STING) (Sixt *et al*. 2017; Weber *et al*. 2017), giving support to the idea that this cell death was induced as part of a host cell-intrinsic defense response. Cell death induced during infection with these mutants was partially apoptotic and partially necrotic (Sixt *et al*. 2017; Weber *et al*. 2017), yet the exact nature of the death pathway(s) activated remains to be uncovered. Moreover, it is not understood how the above-mentioned Inc proteins can prevent cell death induction. It was proposed that their absence causes inclusion membrane instability, which could enhance the release of PAMPs from the inclusion into the host cell cytosol, where they could be sensed, for instance by STING. Furthermore, because CpoS/CT229 interacts with host Rab GTPases, lack of CpoS/CT229 may influence inclusion membrane stability by affecting *Chlamydia*’s ability to subvert host cellular trafficking processes (Sixt *et al*. 2017; Faris *et al*. 2019). It is possible that cell death induced as consequence of premature inclusion lysis could be mechanistically related to late stage host cell death, which is also preceded by inclusion rupture (Hybiske and Stephens 2007; Kerr *et al*. 2017) (Fig. 7).

More recently, a genetic screen in *C. muridarum* uncovered several mutants that were more sensitive to IFN-γ-mediated restriction of bacterial growth in mouse cells (Giebel *et al*. 2019). A closer analysis of one mutant, which carried a specific point mutation in the putative Inc protein TC0574, revealed premature inclusion lysis, followed by induction of host cell death (Giebel *et al*. 2019). Moreover, levels of inclusion lysis and cell death were strongly enhanced when IFN-γ or IFN-β were added to the culture (Giebel *et al*. 2019). In contrast to the cell death observed during infection with the above-mentioned *C. trachomatis* Inc mutants (Sixt *et al*. 2017; Weber *et al*. 2017), this inclusion lysis could be blocked by the pan caspase inhibitor Z-VAD-FMK (Giebel *et al*. 2019). Interestingly, inclusion lysis could also be inhibited partially by specific inhibitors of CASP3, CASP8 and CASP9, while exposure to pro-apoptotic stimuli, such as STS or TNFα/CHX, enhanced inclusion lysis in cells infected with the TC0574 mutant (Giebel *et al*. 2019). The mechanism of inclusion lysis and the nature of the cell death program induced during infection with the mutant remain to be determined.

The *C. trachomatis* and *C. muridarum* Inc mutants mentioned above were significantly attenuated in mouse models of genital infection (Sixt *et al*. 2017; Weber *et al*. 2017; Giebel *et al*. 2019). These observations highlight the protective potential of premature host cell death, as well as the importance for *Chlamydia* spp. to have evolved strategies to block this host defense mechanism.

#### Interactions of Chlamydia spp. with necroptotic and the pyroptotic pathways

While necroptosis can be induced downstream of cytokine receptors and immune sensor proteins (Pasparakis and Vandenabeele 2015), a potential role for necroptosis as a defense mechanism during infection with *Chlamydia* spp. is not well explored. It appears that in contrast to its potent anti-apoptotic activities, *C. trachomatis* L2 does not cause a general block in the necroptotic pathway. Indeed, infection failed to block RIPK3 and MLKL activation in response to the necroptosis inducer TSZ (a mixture of TNFα, SMAC mimetic BV6 and Z-VAD-FMK) and even appeared to sensitize host cells to TSZ-induced necrotic death (Sixt *et al*. 2018).

Pyroptosis, a pro-inflammatory form of necrosis activated by inflammatory caspases, is another prominent example of a cell-autonomous defense response that triggers the death of the infected cell (Cookson and Brennan 2001). Numerous studies showed that *Chlamydia* spp. can, typically within few hours after infection, induce activation of canonical inflammasomes, more precisely activation of the NACHT, LRR and PYD domains-containing protein 3 (NLRP3) and absent in melanoma 2 (AIM2) inflammasomes (Abdul-Sater *et al*. 2009, 2010; He *et al*. 2010; Shimada *et al*. 2011; Nagarajan *et al*. 2012; Itoh *et al*. 2014; Finethy *et al*. 2015; Webster *et al*. 2017). Consequently, infection can lead to the activation of CASP1 and secretion of the pro-inflammatory cytokines IL-1β and IL-18 (Ojcius *et al*. 1998; Lu, Shen and Brunham 2000; Cheng *et al*. 2008; Abdul-Sater *et al*. 2009; Prantner *et al*. 2009; Abdul-Sater *et al*. 2010; He *et al*. 2010; Shimada *et al*. 2011; Nagarajan *et al*. 2012; Itoh *et al*. 2014; Finethy *et al*. 2015; Chen *et al*. 2017; Webster *et al*. 2017). Most of these studies investigated the interaction of *Chlamydia* spp. with human or murine monocytes or macrophages (Ojcius *et al*. 1998; Cheng *et al*. 2008; Prantner *et al*. 2009; Abdul-Sater *et al*. 2010; He *et al*. 2010; Shimada *et al*. 2011; Nagarajan *et al*. 2012; Itoh *et al*. 2014; Finethy *et al*. 2015; Chen *et al*. 2017; Webster *et al*. 2017). Few studies demonstrated that inflammasome activation also occurs in epithelial cells, such as in HeLa cells, although with slower kinetics (Lu, Shen and Brunham 2000; Cheng *et al*. 2008; Abdul-Sater *et al*. 2009). *Chlamydia pneumoniae* was a more potent inducer of inflammasome and CASP1 activation when compared with *C. trachomatis* (D or L2), *C. caviae* and *C. muridarum* (He *et al*. 2010; Itoh *et al*. 2014). A subset of the above-mentioned studies also reported induction of pyroptosis (Finethy *et al*. 2015; Chen *et al*. 2017; Webster *et al*. 2017). Moreover, there is evidence that *C. trachomatis* and *C. muridarum* also induce the non-canonical inflammasome pathway and that both CASP1 and CASP11 contribute to pyroptotic death in infected murine bone marrow-derived macrophages (BMDMs) (Finethy *et al*. 2015; Webster *et al*. 2017).

While *Chlamydia* infection appeared to be self-sufficient in providing both signals required for inflammasome activation, priming of macrophages with extracellular ATP, LPS or IFN-γ enhanced or accelerated the response (Prantner *et al*. 2009; He *et al*. 2010; Finethy *et al*. 2015). Moreover, STING-dependent induction of type I interferon (IFN) production and autocrine IFN signaling were also implicated in enhancing inflammasome activation and pyroptosis in *Chlamydia*-infected cells (Webster *et al*. 2017). This suggests that *Chlamydia* virulence strategies that aim at blocking STING activation, or at dampening the IFN response by other means, may also help the pathogen to limit pyroptosis. Moreover, a recent study provided evidence that the unique structure of chlamydial LPS reduces its potential to activate the non-canonical inflammasome pathway (Yang *et al*. 2019). More precisely, it was shown that in contrast to *E.coli* LPS, LPS from *C. trachomatis* had a very low ability to induce activation of CASP1 and IL-1β release (through CASP11-mediated inflammasome activation) and pyroptosis, when transfected into murine BMDMs (Yang *et al*. 2019). The low toxicity of *C. trachomatis* L2 LPS upon transfection was confirmed in a more recent study (Wang, Rockey and Dolan 2020).

The role of pyroptosis in controlling *Chlamydia* infection is not well understood and its investigation is complicated by the fact that inflammasome activation has multiple additional effects on the immune response, such as via the secretion of pro-inflammatory cytokines. Furthermore, some studies suggested that CASP1-deficiency or inhibition in infected host cells can negatively affect intracellular chlamydial growth (Abdul-Sater *et al*. 2009; Christian *et al*. 2011; Itoh *et al*. 2014).

It should be noted that the recent observation that *C. trachomatis* can induce minority MOMP in infected cells clearly shows that the role of the apoptotic machinery in immunity against *Chlamydia* can also not be reduced to its pro-death function. Indeed, it was shown that minority MOMP can cause DNA damage and mitochondrial damage, which in turn may act as danger signals boosting cytokine secretion and cell-autonomous immunity (Brokatzky *et al*. 2019).

#### *Chlamydia*-mediated inhibition of NETosis

NETosis is a special form of regulated cell death that occurs in neutrophils as part of the host defense. It enables the cells to release so-called neutrophil extracellular traps (NETs), which are large extracellular structures that are composed of proteins and decondensed chromatin (Brinkmann *et al*. 2004). NETs can trap pathogens and block their dissemination. Moreover, NETs contain antimicrobials that can kill pathogens directly (Papayannopoulos 2018). The role of NETs in anti-chlamydial immunity is not well explored. A recent study provided evidence that *C. trachomatis* L2 can block neutrophil activation and hence also NETosis (Rajeeve *et al*. 2018). Mechanistically, this was linked to the *Chlamydia* protease CPAF, which was shown to cleave formyl peptide receptor 2 (FPR2), a surface receptor required for neutrophil activation (Rajeeve *et al*. 2018). The CPAF-deficient mutant was attenuated in a mouse model of infection and this attenuation was not observed in a FPR2-deficient mouse model nor in neutropenic mice (Rajeeve *et al*. 2018). While these data clearly demonstrated the role of neutrophils in anti-chlamydial immunity and the significance of CPAF as virulence factor, further studies are required to clarify the relative role of NETosis compared with other antibacterial neutrophil responses.

## CONCLUSIONS AND PERSPECTIVES

In summary, the interaction of *Chlamydia* spp. (and their relatives) with host cellular survival and death pathways is highly complex and our understanding of the underlying mechanisms and their significance remains scarce despite extensive research progress.

While it is clear that late stage host cell death is an exit strategy that promotes *Chlamydia* spread to formerly uninfected cells, the molecular mechanisms that regulate this important event in *Chlamydia*’s infection cycle remain obscure. It will be important to identify the virulence factors that regulate the execution of bacterial egress and its timing during the development cycle, as well as to clarify the potential roles of host RCD programs. Furthermore, our understanding of the relevance of alternative exit strategies, including their influence on bacterial viability and infectivity, pathogen dissemination, inflammation and tissue damage, and the nature of the host immune response is very limited. Potential effects of phenomena such as bystander cell death and multiplication-independent cytotoxicity should also not be neglected.

Similarly, as we learn more and more about the mechanisms of *Chlamydia* anti-apoptosis, our knowledge about anti-apoptotic *Chlamydia* virulence factors remains scarce and it becomes clear that the function of this virulence trait is not well understood. The number of anti-apoptotic virulence strategies described for *Chlamydia* spp. is vast, yet the relative importance of these strategies is unknown. Moreover, to evaluate the significance of apoptosis inhibition in infected cells, we would need to have a better understanding of the nature and strength of pro-apoptotic signals that infected cells typically encounter *in vivo*. Finally, while recent advances demonstrated the potential of host cell death to serve as host defense response in pathogen restriction, we know little about how these defense programs are trigged and executed by the host or how they are blocked by the pathogen.

Fortunately, it can be predicted that the recently established genetic tools for *Chlamydia* spp. will empower and inspire researchers to apply novel approaches to tackle these questions. For instance, once the molecular basis of bacterial egress is better defined, these tools may be used to directly modify the relative frequency of the distinct exit strategies, such as extrusion vs host cell death, or apoptosis-like vs necrotic host cell death, and to study the consequences for the bacteria and the host in cell culture and *in vivo*. In fact, a recent study reported that a *C. trachomatis* mutant deficient for the Inc CT228 displayed enhanced rates of extrusion and infection with the strain was cleared slower in a murine genital tract infection model (Shaw *et al*. 2018). However, whether these phenotypes are mechanistically linked still needs to be explored.

Interestingly, past research on *Chlamydia* exit and anti-apoptosis has led to partially conflicting findings. It is possible that some reported observations reflect unnatural behavior of immortalized cell lines. Hence, it will be an important task for future research to reassess findings made in cell lines in more natural infection systems, as well as to confirm that similar mechanisms operate in more recent isolates of the bacteria. However, we should also be open for the possibility that some of these discrepancies may reflect true biological differences. For instance, as described above, there is clear evidence that *C. trachomatis* and *C. pneumoniae* use different molecular mechanisms to block host cell apoptosis and it is plausible that similar differences may exist between other species and in the context of exit strategies. Indeed, it is an intriguing possibility that these differences could directly contribute to the distinct disease spectra and tropisms seen among *Chlamydia* spp.

**Figure 8.**
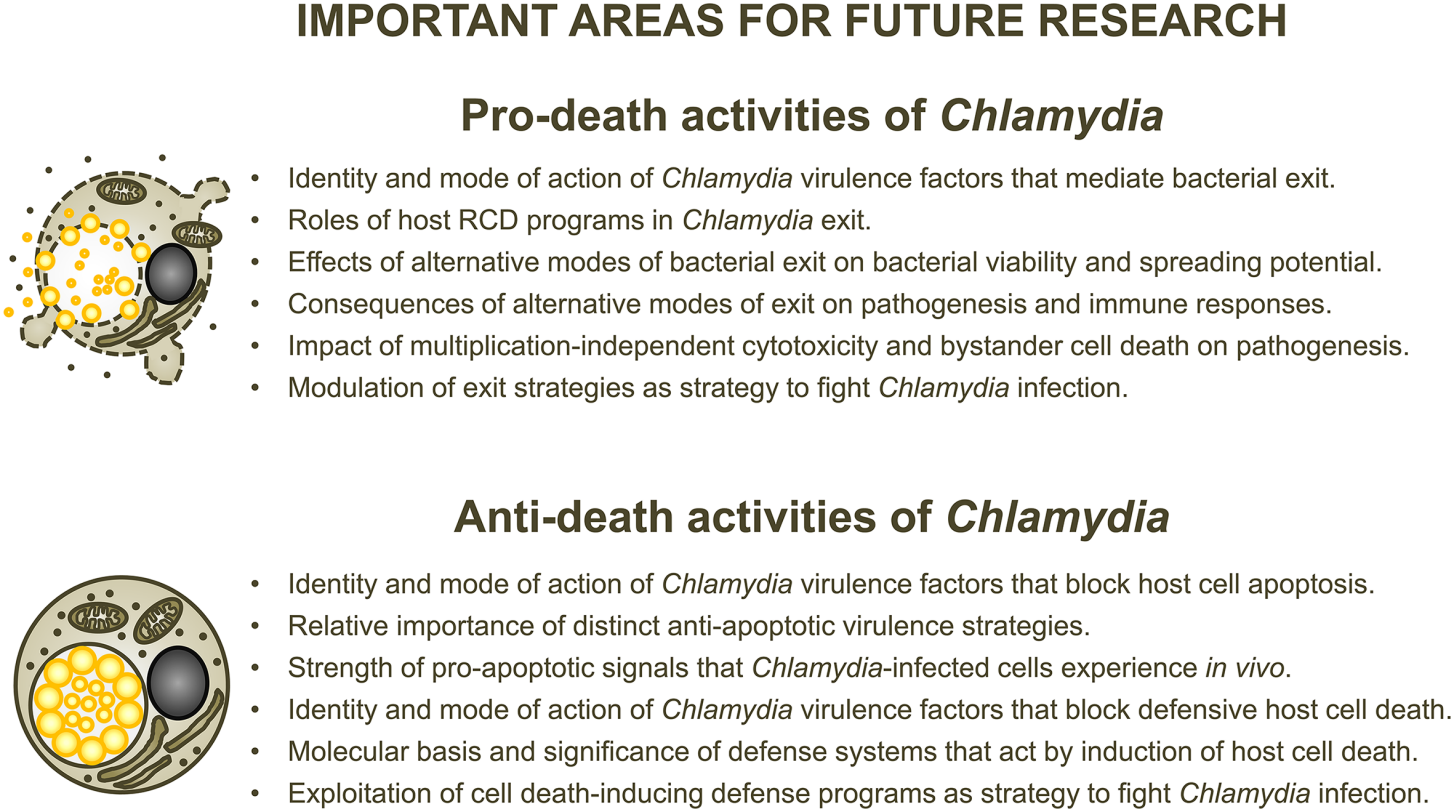
Important areas for future research. This figure gives an overview of highly relevant questions that need to be addressed in depth in the future.

In conclusion, the interaction of *Chlamydia* spp. with host cell death and survival pathways remains an active and stimulating field of research (Fig. 8). A deeper knowledge in this area will be critical for our understanding of *Chlamydia* diseases and anti-chlamydial immunity. Moreover, it is tempting to speculate that it may even inspire novel anti-virulence or host-directed therapeutic strategies. For instance, it may well be possible to fight infectious agents by modulation of pathogen exit strategies or by exploiting host cell-intrinsic defense responses that act by inducing cell death in infected cells.

## ACKNOWLEDGMENTS

I would like to thank the research community for their invaluable contributions to our knowledge, for inspiring discussions and for continuous support and collaboration. I would also like to apologize to all colleagues whose work was not discussed in this review.
